# Functional Characterization of 15 Novel Dense Granule Proteins in Toxoplasma gondii Using the CRISPR-Cas9 System

**DOI:** 10.1128/spectrum.03078-22

**Published:** 2022-12-14

**Authors:** Xiao-Nan Zheng, Jin-Lei Wang, Hany M. Elsheikha, Meng Wang, Zhi-Wei Zhang, Li-Xiu Sun, Xin-Cheng Wang, Xing-Quan Zhu, Ting-Ting Li

**Affiliations:** a Laboratory of Parasitic Diseases, College of Veterinary Medicine, Shanxi Agricultural University, Taigu, China; b State Key Laboratory of Veterinary Etiological Biology, Key Laboratory of Veterinary Parasitology of Gansu Province, Lanzhou Veterinary Research Institute, Chinese Academy of Agricultural Sciences, Lanzhou, China; c Institute of Urban Agriculture, Chinese Academy of Agricultural Sciences, Chengdu, China; d Faculty of Medicine and Health Sciences, School of Veterinary Medicine and Science, University of Nottingham, Loughborough, United Kingdom; e Key Laboratory of Veterinary Public Health of Higher Education of Yunnan Province, College of Veterinary Medicine, Yunnan Agricultural University, Kunming, China; Hubei University of Medicine

**Keywords:** *Toxoplasma gondii*, dense granule proteins, subcellular localization, virulence, cysts

## Abstract

The analysis of the subcellular localization and function of dense granule proteins (GRAs) is of central importance for the understanding of host-parasite interaction and pathogenesis of Toxoplasma gondii infection. Here, we identified 15 novel GRAs and used C-terminal endogenous gene tagging to determine their localization at the intravacuolar network (IVN), parasitophorous vacuole (PV), or PV membrane (PVM) in the tachyzoites and at the periphery of the bradyzoites-containing cysts. The functions of the 15 *gra* genes were examined in type I RH strain and 5 of these *gra* genes were also evaluated in the cyst-forming type II Pru strain. The 15 novel *gra* genes were successfully disrupted by using CRISPR-Cas9 mediated homologous recombination and the results showed that 13 *gra* genes were not individually essential for T. gondii replication *in vitro* or virulence in mice during acute and chronic infection. Intriguingly, deletion of *TGME49_266410* and *TGME49_315910* in both RH and Pru strains decreased the parasite replication *in vitro* and attenuated its virulence, and also reduced the cyst-forming ability of the Pru strain in mice during chronic infection. Comparison of the transcriptomic profiles of the 15 *gra* genes suggests that they may play roles in other life cycle stages and genotypes of T. gondii. Taken together, our findings improve the understanding of T. gondii pathogenesis and demonstrate the involvement of two novel GRAs, TGME49_266410 and TGME49_315910, in the parasite replication and virulence.

**IMPORTANCE** Dense granule proteins (GRAs) play important roles in Toxoplasma gondii pathogenicity. However, the functions of many putative GRAs have not been elucidated. Here, we found that 15 novel GRAs are secreted into intravacuolar network (IVN), parasitophorous vacuole (PV), or PV membrane (PVM) in tachyzoites and are located at the periphery of the bradyzoite-containing cysts. TGME49_266410 and TGME49_315910 were crucial to the growth of RH and Pru strains *in vitro*. Deletion of *TGME49_266410* and *TGME49_315910* attenuated the parasite virulence in mice. However, disruption of other 13 *gra* genes did not have a significant impact on the proliferation and pathogenicity of T. gondii *in vitro* or *in vivo*. The marked effects of the two novel GRAs (TGME49_266410 and TGME49_315910) on the *in vitro* growth and virulence of T. gondii are notable and warrant further elucidation of the temporal and spatial dynamics of translocation of these two novel GRAs and how do they interfere with host cell functions.

## INTRODUCTION

Toxoplasma gondii is an opportunistic apicomplexan parasite which infects a wide range of warm-blood hosts and approximately one-third of the world’s human population (1). In healthy individuals, most infections are asymptomatic; however, severe complications, including encephalitis, retinochoroiditis, and death may occur in immunocompromised individuals, such as cancer patients, HIV patients, and organ transplant recipients (1, 2). Primary T. gondii infection in pregnant women can cause miscarriage and birth defects (1, 2). Considering the lack of effective vaccines against T. gondii and the side effect of current therapeutics, new pharmacological targets and effective vaccines are needed (3).

The ability of T. gondii to invade, replicate, and survive inside virtually all eukaryotic cells is enabled by many effector proteins that the parasite deploys to the host cells, which mediate the invasion and hijack cellular machinery, creating a niche for T. gondii replication and survival (4–8). For example, microneme proteins (MICs) mediate parasite attachment to the host cells (5). Rhoptry neck proteins (RONs) and Rhoptry proteins (ROPs) contribute to the formation of an intracytosolic parasitophorous vacuole (PV) physically separated from the cell cytoplasm by the PV membrane (PVM) (6). After the formation of PV, dense granule proteins (GRAs) are secreted into the PV, PVM, intravacuolar network (IVN) and host cell cytosol or nucleus to enable T. gondii to manipulate the host cell functions and successfully colonize the host cells (7–9). At least 70 GRA proteins have been identified by bioinformatics approaches, organelle strategies or proximity-based protein labeling methods (10–13).

GRAs can modify and allow the PVM more access to the nutrient-rich host cytosol and play roles in overcoming host defense mechanisms (4, 7). For example, GRA3 and GRA5 recruit the host endoplasmic reticulum (ER) (14, 15). GRA7 is associated with IVN uptake of lysosome-derived vesicles and prevents the recruitment of host GTPases to the PVM (16). Other GRAs are necessary for the formation of IVN, including GRA2 and GRA6, which absorb Rab vesicles, host cytosolic proteins, and lipid droplets (16–19). GRA14 and GRA64 interact with the endosomal sorting complex required for transport (ESCRT)-related proteins, facilitating the host cytoplasmic protein uptake (20, 21). Small nutrient molecules are absorbed through membrane pores formed by GRA17 and GRA23 on the PVM (22).

Some GRAs are trafficked across the PVM into the host nucleus cytosol and play important roles in modifying the host signaling pathways, regulation of gene expression, and modulation of host cell immunity (4, 7, 9, 23). For example, GRA16, GRA24, *Toxoplasma* inhibitor of STAT1-dependent transcription (*Tg*IST) and *Toxoplasma* E2F4-associated EZH2-inducing gene regulator (TEEGR) (synonymous with inducer of host cyclin E 1 [HCE1]) are exported beyond the PVM and translocated into host cell nuclei (4, 24–29). GRA18 and MAG1 are localized in the host cell cytosol (4, 30, 31). In addition to the host cell localized GRAs, some PVM GRAs also mediate the host-parasite interaction, such as GRA6, GRA7, GRA12, GRA15 and, GRA60 (4, 32–36). In addition, GRA7 and GRA60 are important virulence factors of T. gondii because they counter the immune-related GTPase (IRG) defense system of host cells (4, 34, 35).

The trafficking and export of GRAs to the PVM, host cytosol, or nucleus are mediated by translocon complex, including several GRAs. For example, by complexing with GRA44, GRA45, TgPPM3C, and MYR4, MYR1 plays an important role in GRAs export beyond the PVM, such as *Tg*IST, GRA16, GRA18, and TEEGR (7). GRA42 and GRA43 mediate the trafficking of GRA17, GRA23, and GRA35 to the PVM (37). GRA45 possesses a small heat shock protein (sHSP) domain that is necessary for the translocation of GRA5 and GRA23 to the PVM (38). Combined with GRA45, GRA44 is involved in the export of GRA16 and GRA24 to the host nucleus (39, 40).

Considering the diverse and essential roles of GRAs in mediating the interaction between the host cell and the parasite, identifying novel GRAs and characterizing their involvement in T. gondii pathogenesis is important. Many putative GRAs have been identified in T. gondii via hyperplexed localization of organelle proteins by isotopic tagging (hyperLOPIT) combined with proteomics (10); however, the functions of most GRAs are still unknown. To understand the roles of novel GRAs in the pathogenesis of T. gondii, we used CRISPR-Cas9 system to elucidate the function of the 15 novel GRA proteins. The localization of 15 novel GRAs in tachyzoites and bradyzoites of the RH strain were investigated by using C-terminal endogenous tagging and immunofluorescence assay (IFA). Following the successful deletion of 15 *gra* genes in the RH strain and 5 *gra* genes in the Pru strain, we investigated the effects of *gra* deletion on the lytic cycle *in vitro* and parasite virulence in acute and chronic infection *in vivo*. Our data indicated that two novel GRAs (TGME49_266410 and TGME49_315910) play crucial roles in the growth and virulence of T. gondii.

## RESULTS

### Fifteen novel GRAs are secreted into the IVN, PV, or PVM in the tachyzoites and are localized to the cyst wall or matrix in the bradyzoites.

We investigated 15 *gra* genes (Table 1), designated as dense granule proteins by hyperLOPIT (10). To examine the localization of these 15 GRAs in the tachyzoites and bradyzoites, epitopes tagged with 6×hemagglutinin (6×HA) were introduced into the C-terminal endogenous gene by homologous recombination and confirmed by PCR and DNA sequencing (see Fig. S1A in the supplemental material). Western blotting of the 15 GRAs extracted from tachyzoites verified the insertion of the endogenous epitope tags and showed the expression of the 15 GRAs in T. gondii RH tachyzoites (Fig. S1B). Most of the GRA bands in Western blotting were consistent with the predicted size found in TOXODB (http://toxodb.org). However, the specific band of TGME49_279350 was smaller than the predicted size, while the bands of TGME49_244530 and TGME49_258462 were bigger than those predicted in TOXODB. TGME49_313440 and TGME49_204320 had the predicted size bands and other small bands. These unpredicted bands in Western blotting may be attributed to posttranslational modifications or an inaccurate prediction of the gene model in TOXODB.

**TABLE 1:** Bioinformatic features of 15 novel dense granule proteins (GRAs) of Toxoplasma gondii

Gene ID	Product description	Predicted location	Exons	Phenotype value	Mol wt (kDa)	Predicted signal peptide	TMHMM^*a*^	Acute infection^*b*^	Chronic infection^*b*^
TGME49_313440	Hypothetical protein	Dense granules	1	1.63	35,503	Yes	No	1	17.13
TGME49_247530	Hypothetical protein	Dense granules	1	1.77	22,820	No	Yes	31.47	24.02
TGME49_204320	Hypothetical protein	Dense granules	1	0.84	32,124	No	Yes	3.9	3.72
TGME49_315910	Hypothetical protein	Dense granules	2	–0.32	62,717	No	No	0.91	2.75
TGME49_297900	Hypothetical protein	Dense granules	5	0.94	82,720	No	No	9.7	46.69
TGME49_267740	Hypothetical protein	Dense granules	1	1.78	37,033	No	Yes	12.01	40
TGME49_268970	Hypothetical protein	Dense granules	1	1.19	36,190	Yes	No	2.07	3.29
TGME49_279350	Hypothetical protein	Dense granules	4	1.73	36,639	No	Yes	7.91	0
TGME49_258462	Hypothetical protein	Dense granules	2	1.79	36,469	No	Yes	18.88	7.58
TGME49_214410	Hypothetical protein	Dense granules	1	2.77	62,644	No	Yes	7.1	23.07
TGME49_291630	Hypothetical protein	Dense granules	1	1.35	25,642	No	No	2.65	1.4
TGME49_258458	Hypothetical protein	Dense granules	3	1.47	28,875	No	Yes	10.92	7.59
TGME49_266410	Hypothetical protein	Dense granules	1	–2.47	63,916	No	Yes	3.37	8.99
TGME49_248990	Hypothetical protein	Dense granules	2	1.7	79,090	No	No	29.54	154.41
TGME49_244530	Hypothetical protein	Dense granules	1	2.12	63,997	No	No	20.29	3.64

a Prediction of transmembrane helices was performed using the TMHMM program version 2.0.

b Gene expression levels of fragments per kilobase of exon model per million mapped reads (FPKM) at days 10 and 28 postinfection, representing acute and chronic infection, respectively.

**FIG 1:**
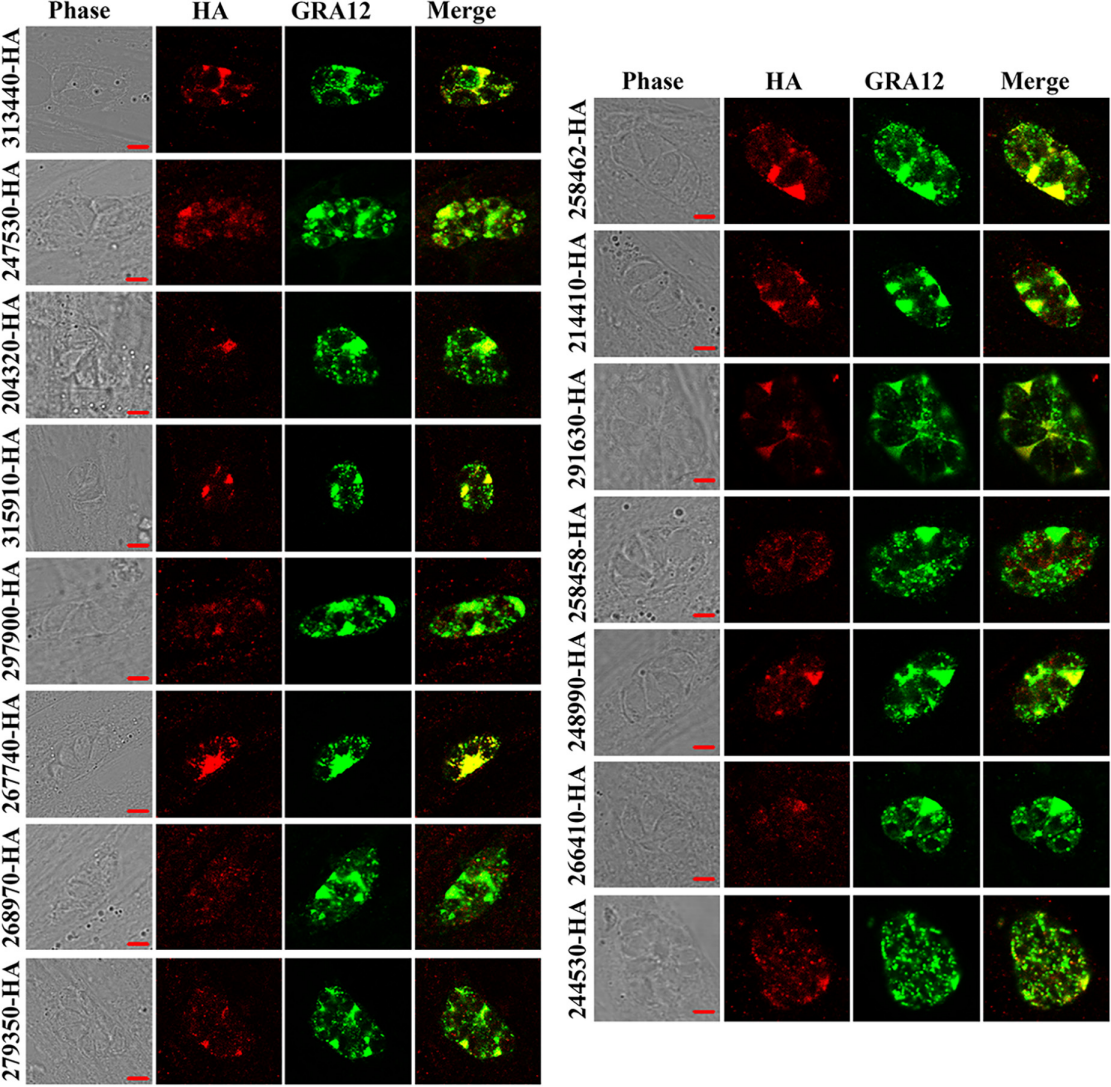
Subcellular localization of 15 GRAs in the tachyzoites of Toxoplasma gondii RH strain. Tachyzoites with HA-tagged GRAs were used to infect HFF cells and stained with antibodies against the GRA12 (green) and HA epitope (red) 24 h postinfection. All 15 GRAs were colocalized or partially colocalized with GRA12. Scale bars, 3 μm.

To verify the subcellular localization of GRAs in T. gondii tachyzoites, human foreskin fibroblast (HFFs) were infected with RH::GRAs-6HA for 24 h, and then examined by confocal microscopy using anti-HA antibodies and rabbit anti-GRA12 antibodies as a GRA marker. As shown in Fig. 1, the 15 predicated GRAs were secreted into the IVN, PV, or PVM, and colocalized or partly colocalized with GRA12 located in the IVN, PVM, and PV (36, 41). The localizations of the 15 novel GRAs are consistent with that of known GRAs (10). These results indicate that these 15 proteins are GRA proteins.

**FIG 2:**
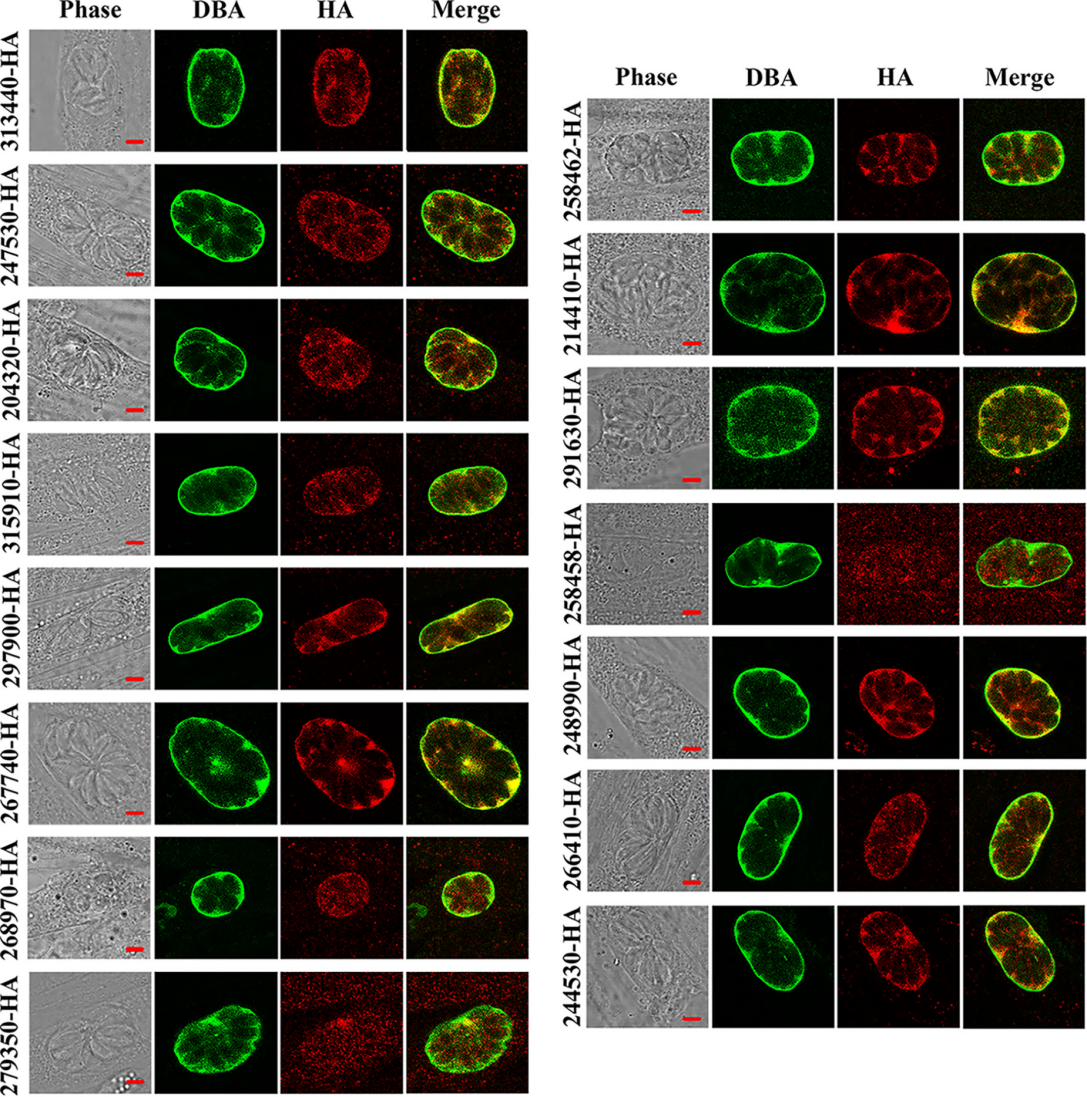
Subcellular localization of 15 GRAs in the bradyzoites of Toxoplasma gondii RH strain. Tachyzoites with HA-tagged GRAs were used to infect HFF cells for 2 h. The infected cells were then cultured under stress condition in alkaline medium for 48 h at 37°C in ambient air. After 2 days, bradyzoites differentiated from tachyzoites were stained with antibodies against HA epitope (red) and DBA-FITC (green). The 15 GRAs were localized to the matrix or wall of the immature bradyzoites-containing cysts. Scale bars, 3 μm.

To further characterize the location of GRAs in the *in vitro*-induced cyst, tachyzoites were allowed to differentiate into bradyzoites by shifting the pH of medium to 8.2 for 2 days. Cysts were detected by DBA staining, which specially recognizes N-acetylgalactosamine on the bradyzoite-specific cyst wall (42, 43), and targeted GRA proteins were detected by anti-HA antibody. By 48 h postdifferentiation (44, 45), 15 GRAs were localized to the cyst wall or matrix, which were colocalized or partly colocalized with DBA (Fig. 2). Combined with previous reports (42) showing that GRAs are secreted into the cyst matrix or wall, our results suggest that these 15 GRAs may play roles in the development of the cysts.

### Fifteen novel *gra* genes are successfully disrupted in the type I T. gondii RH strain.

**FIG 3:**
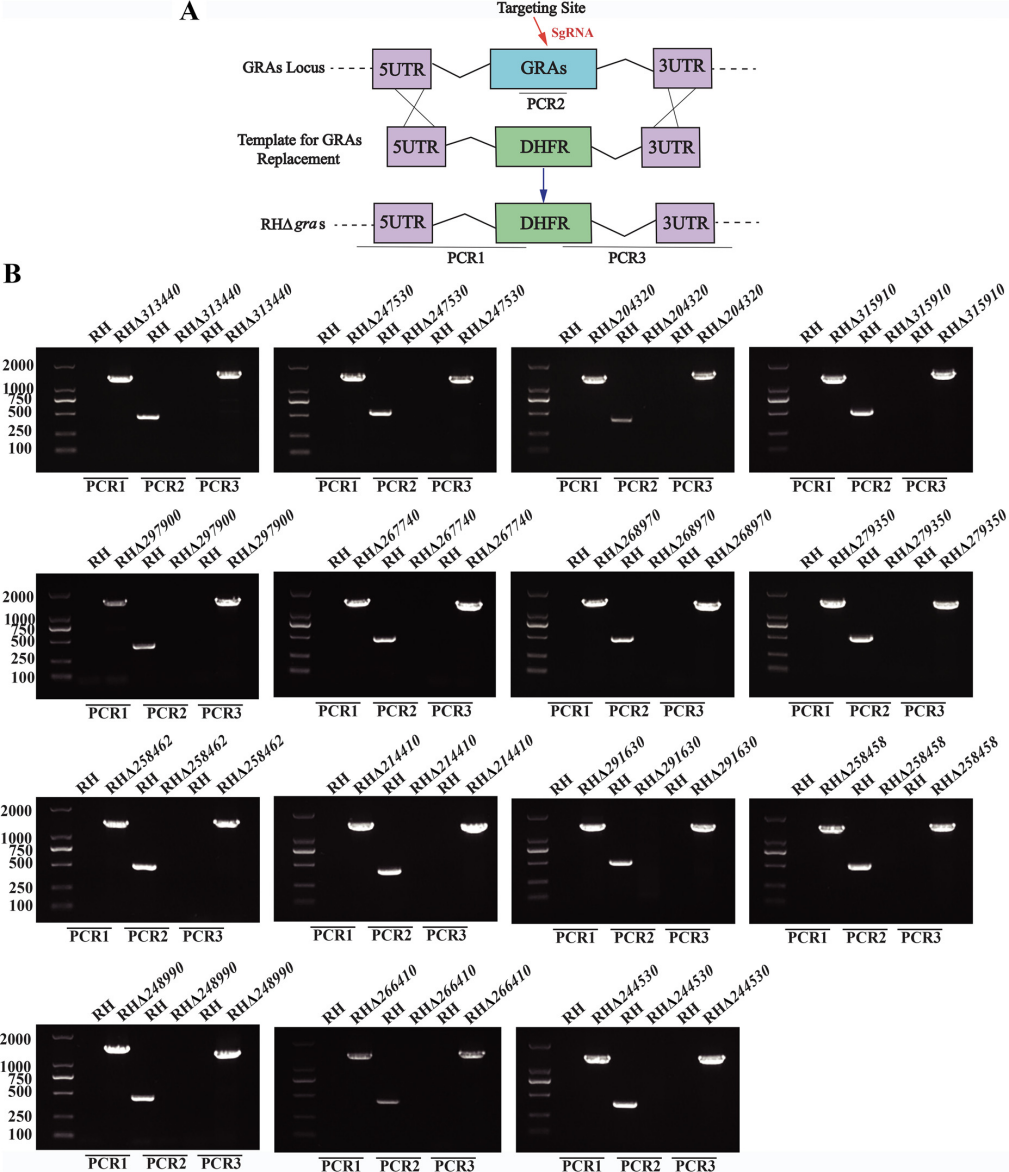
Construction of 15 RHΔ*gra* strains. (A) Schematic illustration showing the deletion of *gra* genes using the CRISPR-Cas9 approach. (B) Identification of 15 RHΔ*gra* strains by diagnostic PCRs. PCR1 and PCR3 were designed to detect the insertion of a homologous fragment into the 5′ and 3′ of *gra* genes, respectively. PCR2 assay was used to detect the successful replacement of *gra* genes by DHFR fragment.

To determine the function of the GRAs, each *gra* locus was disrupted using the CRISPR-Cas9 system (Fig. 3A). A dihydrofolate reductase (DHFR) selectable marker surrounded with the *gra* gene flanking regions (5UTR-DHFR-3UTR) was used to replace the GRA-coding sequence. The CRISPR-Cas9 plasmid targeting each *gra* gene and the corresponding homologous drug-selective fragment were cotransfected into freshly egressed tachyzoites. To obtain single clones, transfectants were diluted using a 10-fold gradient dilution method after selection with pyrimethamine. Diagnostic PCRs confirmed the disruption of each *gra* gene (Fig. 3B). The small fragments (~500 bp) of the *gra* loci were amplified using diagnostic PCR2 which were not amplified in the knockout strains. The successful replacement of each *gra* gene was verified by diagnostic PCR1 and PCR3, in which ~1,500 bp fragments were amplified in the mutant strains but were not amplified in the wild-type strain. The results of diagnostic PCRs demonstrated that all 15 *gra* genes were successfully disrupted in the RH strain by CRISPR-Cas9-mediated homologous recombination (Fig. 3B).

### Loss of TGME49_266410 or TGME49_315910 impairs the intracellular replication in the RH strain.

**FIG 4:**
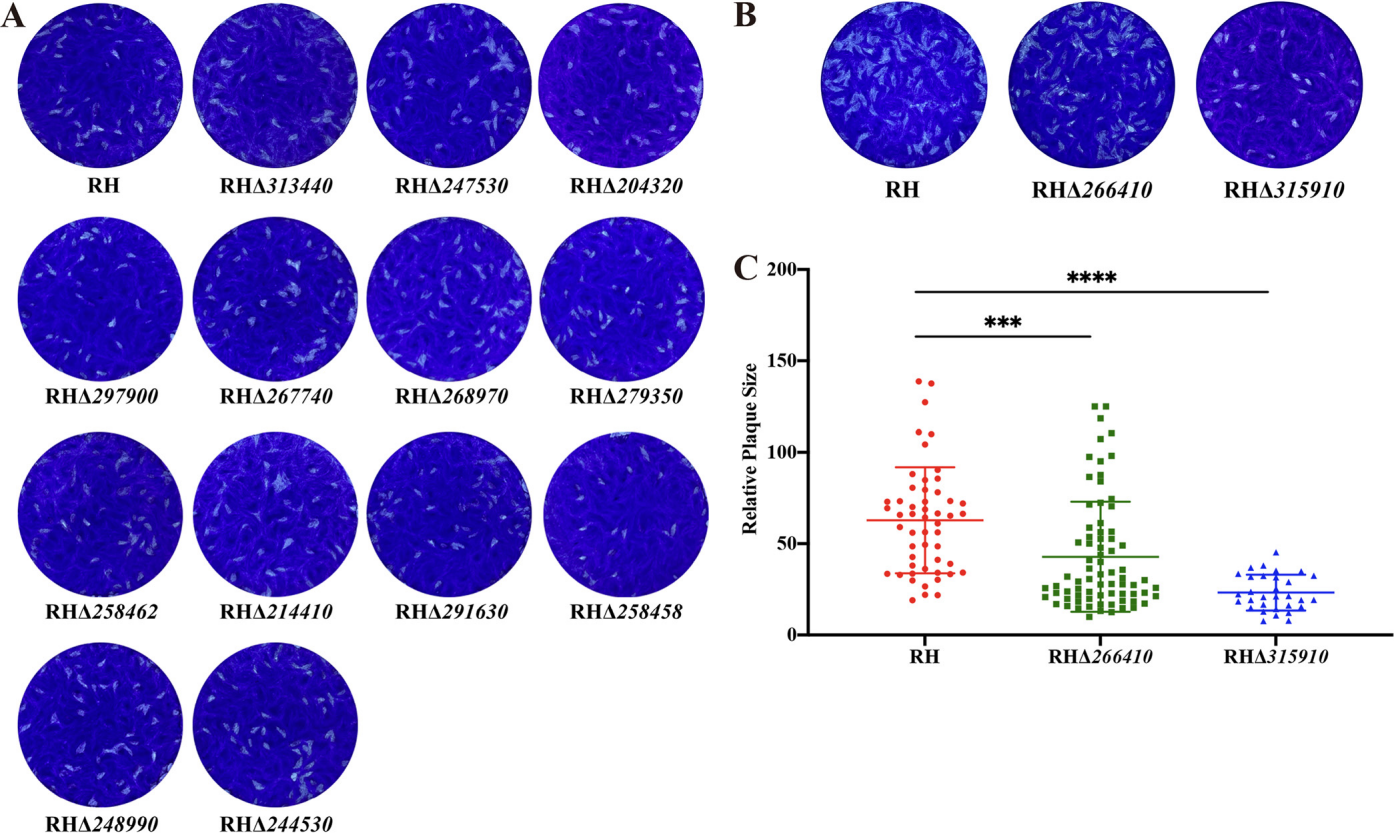
Assessment of the lytic cycle of the 15 RHΔ*gra* and wild-type T. gondii strains. (A) Deletion of 13 *gra* genes had no impact on the lytic cycle in the type I RH strain as determined by the number and size of plaques formed 7 days postinfection of HFFs. (B) The plaques formed in HFFs by the tachyzoites of RH, RHΔ*266410*, and RHΔ*315910* strains. (C) The number and size of plaques produced by RHΔ*266410* and RHΔ*315910* strains, both showed a significant reduction compared to the RH strain (***, *P* < 0.001; ****, *P* < 0.0001). Each plaque was represented by a symbol.

Plaque assays were conducted to determine the effect of *gra* deletion on the lytic cycle of tachyzoites, which represents several processes, including motility, invasion, intracellular growth, and egress (46). HFF cell monolayers grown in 12-well tissue culture plates were infected by freshly egressed tachyzoites of the RHΔ*gra* and wild-type strains. After 7 days of incubation, cells were stained by crystal violet to visualize the plaques. As shown in Fig. 4A, no significant difference was detected in the number and size of plaques between 13 RHΔ*gra* strains (RHΔ*313440*, RHΔ*247530*, RHΔ*204320*, RHΔ*297900*, RHΔ*267740*, RHΔ*268970*, RHΔ*279350*, RHΔ*258462*, RHΔ*214410*, RHΔ*291630*, RHΔ*258458*, RHΔ*248990*, and RHΔ*244530*) and the wild-type strain. However, deletion of *TGME49_266410* and *TGME49_315910* resulted in a significant reduction in the plaque formation (Fig. 4B and C).

**FIG 5:**
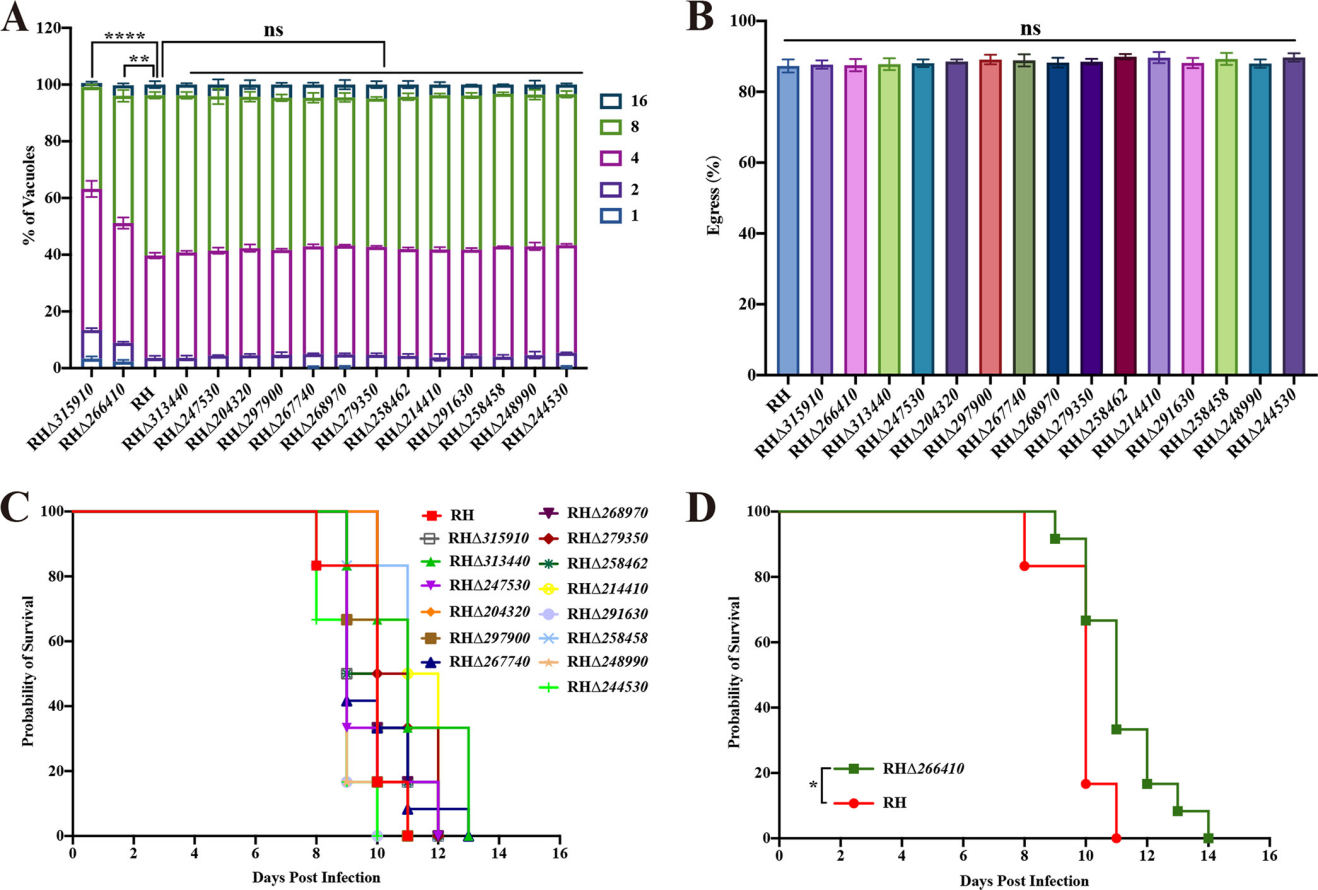
Intravacuolar replication, egress, and survival of Kunming mice infected by the RH strain and the 15 RHΔ*gra* strains. (A) The intracellular growth of the indicated T. gondii strains at 24 h postinfection of HFFs was determined by counting the number of parasitophorous vacuoles (PVs) containing 1, 2, 4, 8, or 16 tachyzoites. At least 200 PVs of each strain were examined in three independent experiments. Intravacuolar replications of only RHΔ*315910* and RHΔ*266410* were significantly slower than that of RH (**, *P < *0.01; ****, *P < *0.0001; ns, nonsignificant). (B) No statistically significant difference was detected in tachyzoite egress between all the mutant strains and the wild-type RH strain. (C) Survival curves of Kunming mice show no significant difference in the RH infected group and 14 RHΔ*gra* infected group. Mice survival was monitored in each group (*n *= 6) after intraperitoneal (i.p.) inoculation with 100 tachyzoites. (D) Mice infected with RHΔ*266410* showed a median time to the humane endpoint of 11 days compared to 10 days for mice infected by the wild-type RH strain. The difference between survival curves was significant (*, *P < *0.05; Gehan-Breslow-Wilcoxon test).

To further investigate whether the 15 novel GRAs are necessary for parasite replication, HFFs were infected with tachyzoites of the RHΔ*gra* and wild-type strains. The parasite replication rate was monitored by counting the number of tachyzoites per PV in at least 200 PVs at 23 h postincubation by using a fluorescence microscope. No significant changes were observed in the replication rates of 13 RHΔ*gra* strains, whereas RHΔ*266410* and RHΔ*315910* exhibited a significant decrease in the parasite intracellular proliferation (Fig. 5A).

To further characterize the effect of *gra* deletion on the parasite egress efficiency, egress assays were performed. The results showed no significant difference in the egress capacity between the 15 RHΔ*gra* strains and the wild-type RH strain (Fig. 5B).

### The virulence of TGME49_266410-deficient mutant in the RH strain is attenuated *in vivo*.

To investigate the function of GRAs *in vivo*, Kunming mice (6 mice per group) were injected intraperitoneally (i.p.) with 100 tachyzoites of each RHΔ*gra* strain and the wild-type RH strain. The mice were monitored twice daily postinfection and those reaching their humane endpoint criteria were euthanized. The survival rates of all infected mice are shown in Fig. 5C and D. The knockout of 14 *gra*s (*TGME49_313440*, *TGME49_247530*, *TGME49_204320*, *TGME49_315910*, *TGME49_297900*, *TGME49_267740*, *TGME49_268970*, *TGME49_279350*, *TGME49_258462*, *TGME49_214410*, *TGME49_291630*, *TGME49_248990*, *TGME49_244530*, and *TGME49_258458*) had no statistically significant effect on the mouse survival time (Fig. 5C). However, mice infected with tachyzoites of the TGME49_266410 mutant strain had slightly longer survival time compared with that of the wild-type RH strain infected group in acute infection (*P < *0.05; Fig. 5D).

### TGME49_266410 and TGME49_315910 are critical for the growth of Pru tachyzoites.

**FIG 6:**
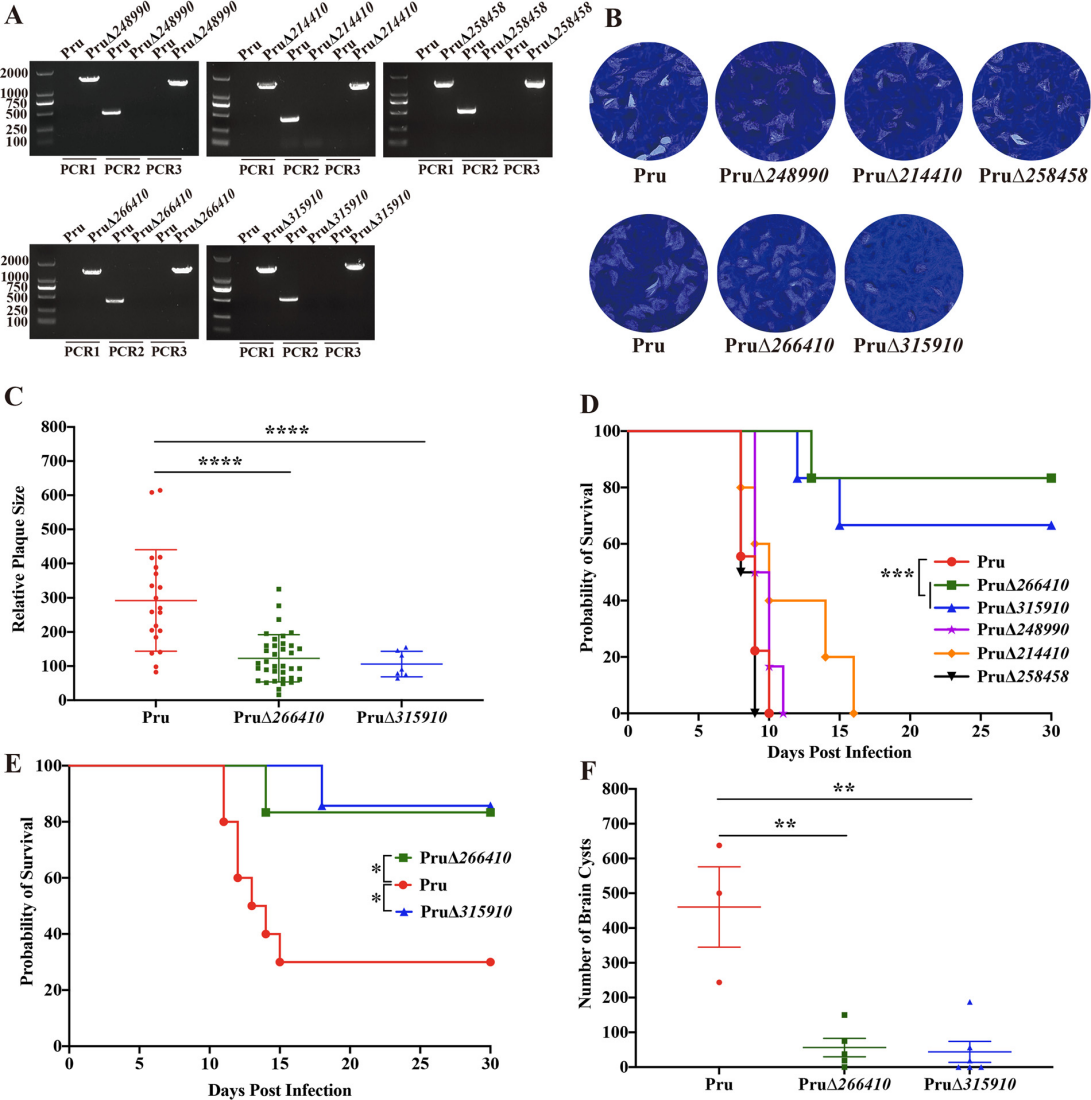
TGME49_266410 and TGME49_315910 contribute to parasite replication in HFF cells and parasite virulence during chronic infection in mice. (A) Identification of 5 PruΔ*gra* strains by diagnostic PCRs. (B) Knockout of *TGME49_248990*, *TGME49_214410*, and *TGME49_258458* had no impact on the lytic cycle in the type II Pru strain, as assessed by the number or size of plaques formed in the HFF cells 12 days postinfection. However, the loss of *TGME49_*266410 and *TGME49_*315910 in the Pru strain affected the plaque formation. (C) The relative size and number of plaques produced by PruΔ*266410* and PruΔ*315910* were significantly reduced compared with that of the Pru strain (****, *P < *0.0001). (D) Survival curves of Kunming mice infected with 5 × 10^4^ freshly egressed tachyzoites of the parental Pru strain and the indicated PruΔ*gra* strains, reaching humane endpoint (***, *P < *0.001). (E) Survival curve of Kunming mice intraperitoneally injected with 200 tachyzoites of Pru, PruΔ*266410*, and PruΔ*315910*, reaching humane endpoint (*, *P* < 0.05). (F) The brain cyst burden in mice infected by 200 tachyzoites of PruΔ*266410* or PruΔ*315910* significantly decreased compared with that of the Pru strain (**, *P* < 0.01).

While 15 *gra* genes were successfully deleted and their functions were investigated in the type I strain (RH), five of the 15 *gra* genes were selected to examine the effect of their deletion on the infectivity of the type II strain (Pru). We chose TGME49_266410 and TGME49_315910 genes because their deletion impacts the parasite fitness, and they have low fitness scores assigned through a genome-wide CRISPR/Cas9 knockout study (47). We also chose TGME49_248990 whose expression is high in bradyzoites and thus more likely to play a role in the Pru strain which forms bradyzoite-containing cysts. Given TGME49_258458 interaction with GRA44, which interacts with MYR1 for delivering T. gondii effector proteins to the host cell (39), we hypothesized that TGME49_258458 may play a role in the Pru strain. TGME49_214410 was randomly selected as a control. As shown in Fig. 6A, five Pru knockout strains (PruΔ*266410*, PruΔ*315910*, PruΔ*248990*, PruΔ*258458*, and PruΔ*214410*) were successfully generated and verified by PCR assays. We performed a plaque assay to examine whether these five novel GRAs play any roles in the lytic cycle of the Pru strain. The deletion of *TGME49_248990*, *TGME49_214410*, and *TGME49_258458* did not affect the propagation of Pru tachyzoites *in vitro* (Fig. 6B). However, the deletion of *TGME49_266410* or *TGME49_315910* significantly reduced the size and number of plaques, in agreement with the results observed in the RH knockout strains (Fig. 6B and C).

### Disruption of *TGME49_266410* or *TGME49_315910* in the Pru strain reduces brain cyst burden *in vivo*.

To evaluate the role of the selected five GRAs in chronic infection, two doses of PruΔ*gra* tachyzoites were inoculated (i.p.) into mice. As shown in Fig. 6D, mice infected with a high dose (5 × 10^4^ tachyzoites) of PruΔ*248990*, PruΔ*214410*, and PruΔ*258458* took a median of 9 days to reach their humane endpoint, without significant difference with the wild-type strain infected group. However, 83% of mice infected with the same dose of PruΔ*266410* or PruΔ*315910* strain remained alive at 30 days postinfection, which was significantly longer than that observed in the parental Pru strain. In the low dose infection assay (200 tachyzoites), more mice infected with the wild-type Pru tachyzoites reached the humane endpoint criteria earlier than mice infected with the tachyzoites of PruΔ*266410* and PruΔ*315910* strains (Fig. 6E). The number of brain cysts determined 30 days postinfection showed a marked reduction in the cyst burden in the PruΔ*266410-*infected group (median = 56 cysts) and PruΔ*315910*-infected group (median = 51 cysts), compared to the wild-type Pru-infected group (median = 460 cysts) (Fig. 6F). These results indicated that TGME49_266410 and TGME49_315910 are important virulence factors in the Pru strain and play a role in chronic infection.

**FIG 7:**
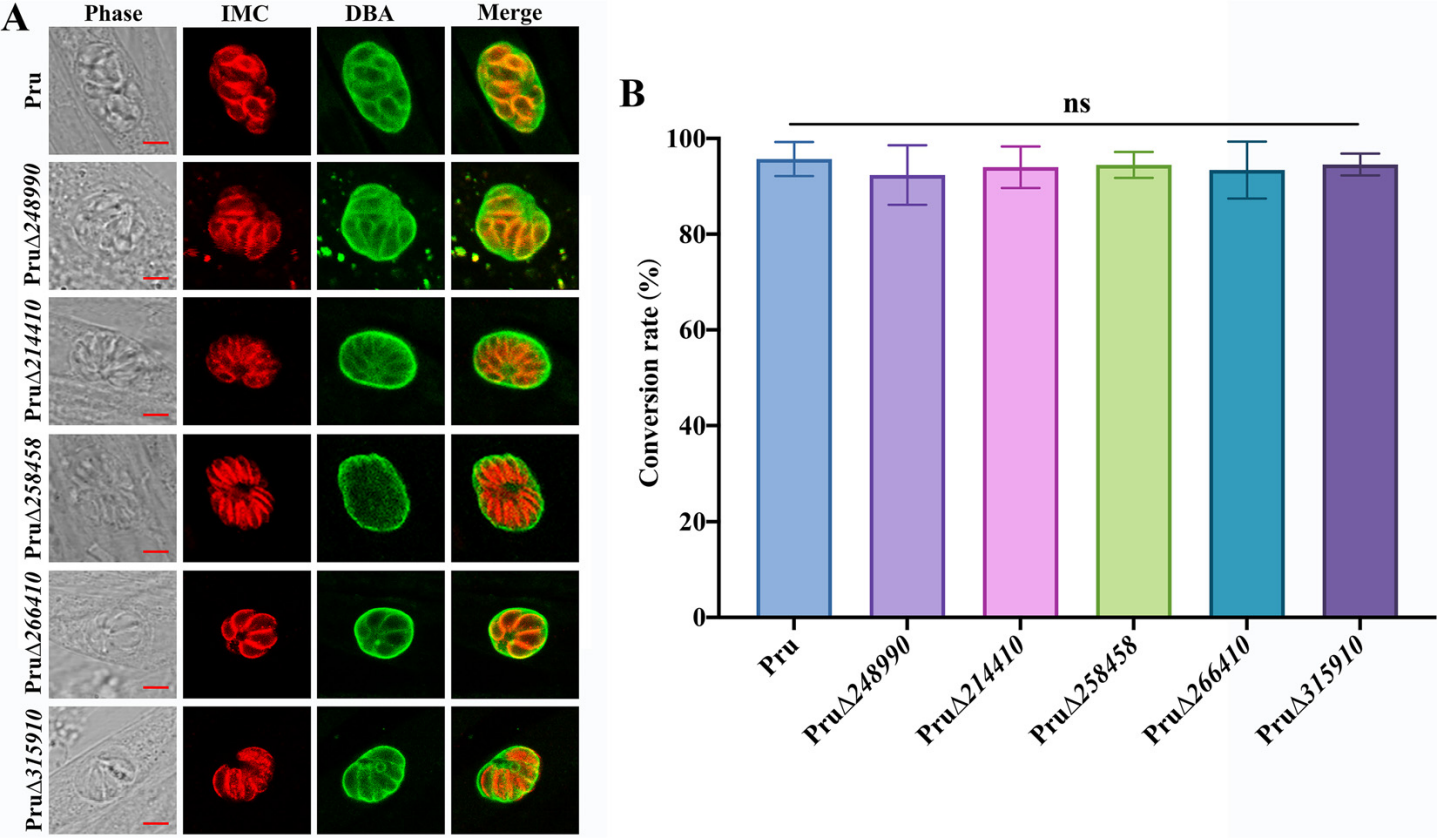
Five GRAs were not essential for the *in vitro* cyst formation in the Pru strain. (A) The cysts formed by Pru and five PruΔ*gra* strains *in vitro*. Confluent HFFs were infected with the Pru and PruΔ*gra* strains, and bradyzoite differentiation was induced using ambient air and pH 8.2 for 2 days *in vitro*. Samples were stained with antibodies against IMC1 (red) and DBA-FITC (green). Scale bars, 5 μm. (B) Cyst conversion rate of the Pru and PruΔ*gra* strains. At least 100 vacuoles in each experiment were counted and classified as cyst wall-positive vacuoles (DBA-positive) or normal vacuoles (DBA-negative). No significant differences in the conversion ratio between the Pru and PruΔ*gra* strains was detected by Student's *t* test.

We further investigated the ability of each of the five GRA mutants (PruΔ*248990*, PruΔ*214410*, PruΔ*258458*, PruΔ*266410*, and PruΔ*315910*) to form bradyzoite-containing cysts *in vitro*. After 2 days differentiation in alkaline pH (pH = 8.2) and ambient air, the expression of the bradyzoite-specific marker DBA was examined to determine the bradyzoite formation (48). Results showed that the *in vitro* cyst-forming ability of these five GRA mutant strains was similar to that of the wild-type parasites (Fig. 7), suggesting that none of these five GRAs are essential for cyst formation *in vitro*.

### Fifteen novel GRAs may play roles in different life cycle stages of T. gondii.

Data about the transcription levels of 13 *gra* genes of different T. gondii lineages, cell cycle phases, life cycle stages, and stage differentiation determined by DNA microarrays was obtained from the TOXODB (https://toxodb.org), except for TGME49_267740 and TGME49_258458 because they had no transcription data in the TOXODB. Among 13 *gra* genes, the transcriptional levels of TGME49_313440 and TGME49_247530 were significantly different in the three T. gondii genotypes (type I, II and III) (see Fig. S2A in the supplemental material). The expression profiles of 13 *gra* genes during the parasite cell cycle phase were also analyzed (Fig. S2B). Most *gra* genes followed a specific cell cycle pattern, with the highest expression level detected at the M and C stages, except six *gra* genes, including *TGME49_315910*, *TGME49_297900*, *TGME49_279350*, *TGME49_258462*, *TGME49_244530*, and *TGME49_291630*, which had the highest expression level at the G stage. During all cell cycle phases, the expression level of *TGME49_244530* was the highest, while *TGME49_315910* and T*GME49_268970* had the lowest expression. Fig. S2C in supplemental material showed the expression profile of all *gra* genes during tachyzoite-bradyzoite differentiation, showing an increase in the expression of some *gra* genes, including *TGME49_313440*, *TGME49_297900*, *TGME49_248990*, and *TGME49_214410*. However, the expression of 5 *gra* genes was decreased, including *TGME49_247530*, *TGME49_268970*, *TGME49_279350*, *TGME49_258462*, and *TGME49_244530*. The remaining four genes (*TGME49_204320*, *TGME49_266410*, *TGME49_315910*, and *TGME49_291630*) were continuously expressed at the differentiated stages. Across the different developmental stages (Fig. S2D), some GRAs, including *TGME49_247530*, *TGME49_297900*, *TGME49_279350*, *TGME49_258462*, *TGME49_248990*, and *TGME49_244530*, were expressed at different levels. The rest of the *gra* genes were consistently expressed.

## DISCUSSION

Despite the significant advances in the knowledge of T. gondii virulence factors, the spectrum and functions of the effector proteins necessary to enable T. gondii infection remain unclear (1, 4, 7–9). GRAs are the most studied and important effector proteins in T. gondii due to their pivotal roles in mediating host-parasite interaction and parasite adaption to the intracellular environment. Most GRAs are localized in the PV, PVM, IVN, and several to the host cytoplasm or nucleus. GRAs localized in the PVM or the host cell affect numerous signaling pathways in the host cell (29) and alter host cell gene expression (24, 28) and immunity (26, 27). The PVM- or IVN-localized GRAs are involved in host cytoskeleton restructuring, nutrient acquisition, and protein translocation. However, the functions of the newly identified putative GRAs are still unknown. Here, we investigated the intracellular localization and functions of 15 novel GRAs in T. gondii type I (RH) and type II (Pru) strains *in vitro* and *in vivo*.

The majority of GRAs are synthesized in the rough ER and then exported from the ER, trafficking through the Golgi to the dense granules, then finally secreted from the parasite to a particular location to perform its function (23). In this study, subcellular localizations of 15 novel GRAs were investigated in the tachyzoites and bradyzoites of the T. gondii RH strain. We found that all 15 novel GRAs were secreted into the IVN, PV, and PVM, and colocalized or partly colocalized with GRA12 in the tachyzoites. Similar findings are reported for other GRAs (11, 38, 49). GRA proteins are also associated with the wall of the bradyzoite-containing cysts to maintain the parasite viability and ability to evade the host immune response (42, 50, 51). In the bradyzoite stage, the 15 novel GRAs were localized in the cyst wall or cyst matrix, which is consistent with the localizations of other GRAs (42, 43, 48).

The posttranslational modification plays crucial roles in the function of some GRAs (52–54). In the present study, the unpredicted bands shown in the Western blotting may be attributed to posttranslational modification, such as procession of aspartyl protease V (ASP5) or phosphorylation by WNG1 (parasite-secreted kinase) (52–55). Except the major bands which matched the predicted size, the other bands of TGME49_313440 and TGME49_204320 detected by Western blotting may be attributed to protein degradation or the processing of TGME49_204320 by ASP5, considering that this putative protein harbors an arginine-arginine-leucine (RRL) motif termed TEXEL (T. gondii export element) (52, 53, 55). The phosphorylation of TGME49_244530 by WNG1 (parasite-secreted kinase), reported previously (54), may cause the bigger bands observed in the Western blotting. The unpredicted bigger band of TGME49_258462 may be caused by posttranslational modifications or an imprecise gene prediction in TOXODB (40). Apart from the major bands, there were other bands in the Western blotting of TGME49_258462. Given that two RRLs were predicted in TGME49_258462, the other bands that are detected in the Western blotting may be caused by the effect of the cleaving of ASP5 (52, 53, 55). Further experiments are needed to verify whether posttranslational modifications are the underpinning mechanisms for these observations.

Like most GRAs that are not essential for the parasite growth *in vitro* (56), CRISPR-Cas9-mediated knockout of 13 *gra* genes had no significant impact on the parasite replication ability, as indicated by the limited differences in the size and number of plaques formed in HFFs by the knockout and wild-type strains, and the insignificant difference in intracellular replication between the 13 *gra* knockout strains and the wild-type strains. This result suggests that none of these 13 novel GRA proteins was individually essential for the parasite survival *in vitro*, which was consistent with the high CRISPR fitness score (47). However, these proteins may play a role in the parasite fitness in other intracellular environment, such as interferon gamma-activated host cells (36, 38).

On the other hand, some GRAs play important roles in T. gondii. Disruption of these GRAs impair the parasite growth, including GRA17 (22, 57), GRA39 (11), GRA41 (58), GRA44 (39), and PPM3C (59). GRA17 along with GRA23 influence PVM permeability and transportation of small molecules (22). GRA41 is critical for regulating calcium homeostasis and egress (58). GRA44 and PPM3C mediate the effector export (39, 59). In the present study, deletion of *TGME49_266410* and *TGME49_315910* in both RH and Pru strains resulted in a marked growth defect of tachyzoites, suggesting that TGME49_266410 and TGME49_315910 play roles in T. gondii propagation. Whether these effects are due to the involvement of TGME49_266410 and TGME49_315910 in the uptake or trafficking of nutrients like GRA17 and GRA23, or protein export like GRA44 and PPM3C, remains to be investigated.

Some GRAs contribute to parasite virulence in mice (11, 22, 38, 57, 59, 60). This effect was also observed in TGME49_266410 in the present study. Disruption of *TGME49_266410* significantly attenuated the virulence of both RH and Pru strains. The reduction in virulence of the knockout strains is likely a consequence of a slow growth of the mutant strain in mice. In contrast, disruption of *TGME49_315910* did not attenuate the virulence of the RH strain; however, it attenuated that of the Pru strain, which is likely to be related to the high virulence of the RH strain (1, 2). The activities of some GRAs have been shown to be strain-dependent (32). Thus, the function of TGME49_315910 might be genotype/strain-specific, given that there is a difference in six amino acids in TGME49_315910 between type I (GT1 strain) and type II (ME49 strain) (http://toxodb.org).

In chronic infection, PruΔ*266410* and PruΔ*315910* had significantly formed fewer brain cysts in mice. Considering the *in vitro* growth kinetics showing significant growth defect in PruΔ*266410* and PruΔ*315910*, the reduced cyst-forming ability of PruΔ*266410* and PruΔ*315910* might be caused by elimination of most tachyzoites inoculated (i.p.) into the mice, although some of the tachyzoites managed to arrive to the brain and form cysts. The exact mechanism by which TGME49_266410 and TGME49_315910 affects the parasite propagation and virulence remains to be investigated. Although PruΔ*266410* and PruΔ*315910* showed attenuated virulence, they can form brain cysts in mice and are thus not promising vaccine candidates against toxoplasmosis. However, *TGME49_266410* and *TGME49_315910* could still be great candidate genes to generate double or triple gene knockout mutants as live-attenuated vaccines.

Transcriptome data available in the TOXODB showed that the expression patterns of *gra* genes vary by different T. gondii genotypes, cell cycle phases, life cycle forms, and bradyzoite differentiation. The different expression of *TGME49_313440* and *TGME49_247530* in different T. gondii genotypes indicate that they may have strain-specific roles like GRA15 (32). Several GRAs are upregulated in the bradyzoites and play an important role in the establishment or maintenance of cysts in the mouse brain, such as GRA55 (12, 48, 50, 51). Among the 15 characterized GRAs, *TGME49_248990* had the highest expression in bradyzoites. However, disruption of this *gra* gene did not change the bradyzoite differentiation rate *in vitro*. Although 13 GRA proteins were not involved in the replication and infectivity of T. gondii, they may play roles in the other developmental stages of this parasite.

In conclusion, our data revealed the roles of two novel GRAs TGME49_266410 and TGME49_315910 in T. gondii virulence. Further investigations are needed to unravel the molecular mechanisms and kinetics of translocation, and their molecular interaction with host cell organelles, all are important elements in understanding T. gondii manipulation of host cell machinery. Our study, along with others, show that GRAs are key virulence factors utilized by T. gondii to facilitate infection and colonization of the host cells, and provide possible targets for the development of novel therapeutics for T. gondii.

## MATERIALS AND METHODS

### Host cell and parasite culture.

Human foreskin fibroblast (HFF) cells (American Type Culture Collection; ATCC SCRC-1041) were cultured in Dulbecco’s Modified Eagle medium (DMEM) supplemented with 10% fetal bovine serum (FBS), 10 mM HEPES (pH 7.2), 100 U/mL penicillin, and 100 μg/mL streptomycin, as described previously (36). Cultured HFFs were maintained in a humidified atmosphere containing 5% CO_2_ at 37°C. The tachyzoites of T. gondii strains, including RHΔ*ku*80, PruΔ*ku*80, and *gra* knockout strains, were maintained in confluent HFF monolayers under the same conditions, except that the concentration of FBS was reduced to 2%. HFF cells heavily infected by tachyzoites were scraped off and passed through 27-gauge needles. The released tachyzoites were purified using a 5 μm Millipore filter, counted using a hemocytometer, and used for the assays described below.

### Generation of GRA knockout parasite strains.

Selected *gra* genes were disrupted by the CRISPR-Cas9 mediated homologous recombination as described previously (36, 61). For construction of a knockout plasmid of each *gra* gene, we used a template plasmid, pSAG1:CAS9-U6-SgUPRT, expressing CAS9 and a single guide RNA targeting the UPRT in T. gondii, to replace the UPRT gRNA with the specific gRNA targeting each *gra* gene. A drug-selective plasmid was constructed by fusing the 5′ and 3′ homologous arms of each *gra* gene amplified from T. gondii genomic DNA, the DHFR fragment amplified from pUPRT-DHFR-D plasmid, and the pUC19 fragment amplified from plasmid pUC19 using the multifragment cloning method by a CloneExpress II one-step cloning kit (Vazyme). This drug-selective plasmid, validated by sequencing, was used as a template to amplify the fragment of 5UTR-DHFR-3UTR which was then extracted using a gel extraction kit (Omega). The knockout plasmids, validated by sequencing, were collected by Endo-free plasmid DNA minikit (Omega). The specific knockout plasmid (~35 μg) and corresponding homologous drug-selective fragment (5UTR-DHFR-3UTR; ~20 μg) were cotransfected into freshly egressed tachyzoites by electroporation (62). The *gra*-knockout transfectants were obtained by selection with 3 μM pyrimethamine, and the single clones were obtained by using 96-well tissue culture plates and modified limiting dilution. The confirmation of *gra*-knockout strains was carried out by PCR assays (Fig. 3A). All the primers used to construct the RHΔ*gras* are listed in Table S1 in supplemental file 1.

### Endogenous C-terminal epitope tagging.

For C-terminal endogenous tagging of *gra* genes, a specific CRISPR plasmid targeting the locus near the STOP codon of each *gra* gene was obtained, and the homologous fragment containing 6 × hemagglutinin (6×HA) and DHFR was amplified using p6×HA-LIC-DHFR as a template and a pair of specific primers. One of the primers was designed with 42 bp of the 3′ region of *gra* gene without a STOP codon, and the other primer was designed with 42 bp of the *gra* gene just after the corresponding SgRNA. The successfully sequenced plasmid (~35 μg) and the corresponding purified fragment (~20 μg) were cotransfected into the tachyzoites of RHΔ*ku*80 strain. After drug selection in 96-well tissue culture plates, independent clones were confirmed by sequencing, PCRs, IFA, and Western blotting. Primers used for the generation of epitope-tagged strains are listed in Table S2 in the supplemental material.

### Induction of bradyzoite differentiation.

The tachyzoites of T. gondii were differentiated into bradyzoites *in vitro*, as previously described (44, 45). The tachyzoites of strains with C-terminal HA-tagged GRA, the PruΔ*gra* or wild-type Pru strains were used to infect confluent HFF cells cultured on coverslips at the bottom of 12-well culture plates. The infected HFF cells were washed 2 h after infection using the differentiation medium with pH 8.2 and incubated at 37°C in ambient air. To maintain the alkalinity of the medium, the differentiation medium was replaced daily. The samples were analyzed by IFA after 48 h postdifferentiation (44, 45). The *Dolichos biflorus* agglutinin (DBA) positive vacuoles were assigned as bradyzoite-containing cysts and the percentage of cyst differentiation was calculated based on the results obtained from three independent experiments.

### Detection of GRA proteins by Western blotting and immunofluorescence.

For detection of GRAs and verification of the success of C-terminal tagging, extracellular tachyzoites were collected, centrifuged, and washed with cold phosphate-buffered saline (PBS) twice (10 min, 1,000 × *g*). The purified tachyzoites were lysed by using RIPA lysis buffer on ice for 1 h. The supernatant was collected after centrifugation of the lysates and was blended with 4 × sample loading buffer and boiled for 15 min at 100°C. These samples were analyzed by SDS-PAGE and subsequently transferred to polyvinylidene fluoride (PVDF) membrane by wet electroblotting, as described previously (36). For Western blotting, the primary antibodies were rabbit antialdolase (at 1:500), and rabbit anti-HA (at 1:1,000); the secondary antibody was goat antirabbit (at 1:5,000). Antibodies were obtained from Cell Signaling.

IFA was used to visualize dense granule proteins in tachyzoites and bradyzoites. Tachyzoites were allowed to infect confluent HFF monolayers. After 24 h, the samples were fixed in 4% paraformaldehyde (PFA) for 40 min, permeabilized with 0.2% Triton X-100 for 20 min, and then blocked with 3% bovine serum albumin in PBS. The infected cells were incubated for 2 h at 37°C with primary antibodies, including mouse anti-HA (1:500) and rabbit anti-GRA12 (1:400), and washed five times by cold PBS. Subsequently, cells were incubated for 1 h at 37°C with secondary goat antimouse IgG (H+L) antibodies conjugated with Alexa Fluor 594 (1:500) and donkey antirabbit IgG (H+L) antibodies conjugated with Alexa Fluor 488 (1:500) (36). The samples in which bradyzoites were differentiated were processed with only primary mouse anti-HA antibody (1:500). After five washes, cells were incubated with secondary goat antimouse IgG (H+L) conjugated with Alexa Fluor 594 at a dilution of 1:500 together with DBA-FITC (Vector Laboratories) at a dilution of 1:500 in PBS (36). To test the ability of tachyzoites to form bradyzoite-containing cysts *in vitro*, parasites were stained with rabbit anti-IMC (1:500) and goat antirabbit IgG (H+L) conjugated with Alexa Fluor 594, and bradyzoites were stained with DBA-FITC (1:500) (36). After washing five times, cells were imaged by Leica confocal microscope system (TCS SP8, Leica, Germany).

### Standard *in vitro* plaque assay.

A plaque assay was performed as previously described (36, 63). Briefly, 500 tachyzoites were added to confluent monolayers of HFF cells grown on the surface of 12-well tissue culture plates. After 7 days (for the RHΔ*gra* and wild-type RH strains) or 12 days (for the PruΔ*gra* and wild-type Pru strains) of undisturbed incubation, cells were washed twice with warm PBS, and fixed with 4% PFA for 20 min. Subsequently, cells were stained with 2% crystal violet for 20 min and washed twice with PBS. The number of plaques formed by the replicating tachyzoites was quantified by ImageJ software.

### Intracellular replication and egress.

Confluent HFF cells growing on 6-well tissue culture plates were infected by 10^5^ tachyzoites of T. gondii per well. Infected cells were washed three times with DMEM after 1 h invasion to remove any unbounded tachyzoites. For intracellular replication assay, infected HFF cells were fixed with 4% PFA after 23 h of incubation (63). The cells were stained with mouse anti-SAG1 followed by a secondary goat antimouse IgG conjugated with Alexa Fluor 488. The number of tachyzoites per PV was counted, including 200 randomly selected PVs per sample (36). For the parasite egress experiment, cell culture plates were maintained at 37°C for another 32 to 36 h after removing tachyzoites that remained extracellular. Then, the wells were washed with warm PBS and treated with 3 μM calcium ionophore A23187 (Sigma) diluted in DMEM. Once the egress started, the cultured plates were immediately fixed, and the percentage of egressed or nonegressed PVs was determined, as previously described (63). Three independent experiments were performed for each assay.

### Virulence assessment during acute and chronic infection.

Female Kunming mice (6 to 7 weeks old) were purchased from the Center of Laboratory Animals, Lanzhou Veterinary Research Institute, Chinese Academy of Agricultural Science. All experimental procedures involving the use of mice were reviewed and approved by the Animal Ethics Committee of Lanzhou Veterinary Research Institute, Chinese Academy of Agricultural Sciences (approval no. 2021-008). Every effort was made to reduce the suffering of the animals. Prior to the start of experiment, all mice were habituated for 1 week before allocation to the experimental groups. In acute infection, 100 freshly egressed tachyzoites of the RHΔ*gra* mutant strains and the wild-type RH strain suspended in 200 μL PBS were injected intraperitoneally (i.p.) into mice (6 mice/strain) (64, 65). The viability of the parasites used to infect mice was determined by a plaque assay. For survival rates, the mice were monitored twice daily and the mice that had reached their humane endpoint were immediately euthanized.

We evaluated the roles of five GRAs during chronic infection. Briefly, mice were inoculated by high dose (5 × 10^4^ tachyzoites) or low dose (200 tachyzoites) of five mutant strains (PruΔ*248990*, PruΔ*214410*, PruΔ*258458*, PruΔ*266410*, and PruΔ*315910*) and the wild-type Pru strain by i.p. route. Mice were monitored twice daily for up to one mouth unless they exhibited humane endpoint criteria sooner. We also investigated the cyst-forming ability of the two strains, PruΔ*261410* and PruΔ*315910*, which exhibited the highest survival rates compared to the wild-type Pru strain. In brief, the brains of mice infected by 200 tachyzoites of PruΔ*261410*, PruΔ*315910*, or the Pru strain were collected and homogenized in 1 mL PBS at 30 days postinfection and the number of cysts was counted, as previously described (36).

### Bioinformatics analysis of *gra* genes.

Bioinformatic information on the *gra* genes were obtained from T. gondii genome database (http://toxodb.org). The transcriptional patterns of the main archetypal lineages (genotypes I, II, and III), cell cycle expression profiles (66), different developmental stages (oocysts, tachyzoites, and bradyzoites) (67), and during bradyzoite differentiation (68) were analyzed using the Robust Multiarray Average (RMA) algorithm of the Partek Genomics Suite package (Partek, Inc., St. Louis, MO, USA). The genomic features obtained included the phenotype value, the number of exons, predicted signal peptide, transmembrane domains (TMHMM), and molecular weight.

### Statistical analysis.

Statistical analyses were performed using GraphPad Prism (version 9.0). All data were based on three independent experiments. The results shown are the means ± standard deviations (SD). The significant difference between 2 groups or ≥ 3 groups were determined by two-tailed, unpaired Student’s *t* test, and one-way analysis of variance (ANOVA), respectively. The difference between groups was considered statistically significant when the *P* values were < 0.05.
