# Supplementary material for: Functional Characterization of 15 Novel Dense Granule Proteins in Toxoplasma gondii Using the CRISPR-Cas9 System
Source: Microbiol Spectr. 2022 Dec 14;11(1):e03078-22. doi: 10.1128/spectrum.03078-22 (PMC9927372; doi:10.1128/spectrum.03078-22)
Supplement: Supplemental file 1 — Supplemental material. Download spectrum.03078-22-s0001.pdf, PDF file, 1.3 MB [file spectrum.03078-22-s0001.pdf]

Supplementary material for

Functional Characterization of 15 Novel Dense Granule Proteins in *Toxoplasma gondii* Using the CRISPR-Cas9 System

Xiao-Nan Zheng,[a,b] Jin-Lei Wang,[b,c] Hany M. Elsheikha,[d] Meng Wang,[b,c] Zhi-Wei Zhang,[b] Li-Xiu Sun,[b] Xin-Cheng Wang,[b] Xing-Quan Zhu,[a,e*] Ting-Ting Li[b,c,*]

[a]Laboratory of Parasitic Diseases, College of Veterinary Medicine, Shanxi Agricultural University, Taigu, China

[b]State Key Laboratory of Veterinary Etiological Biology, Key Laboratory of Veterinary Parasitology of Gansu Province, Lanzhou Veterinary Research Institute, Chinese Academy of Agricultural Sciences, Lanzhou, China

[c]Institute of Urban Agriculture, Chinese Academy of Agricultural Sciences, Chengdu, China

[d]Faculty of Medicine and Health Sciences, School of Veterinary Medicine and Science, University of Nottingham, Loughborough, UK

[e]Key Laboratory of Veterinary Public Health of Higher Education of Yunnan Province, College of Veterinary Medicine, Yunnan Agricultural University, Kunming, China

Address correspondence to:

Xing-Quan Zhu: xingquanzhu1@hotmail.com

Ting-Ting Li: litt866@163.com

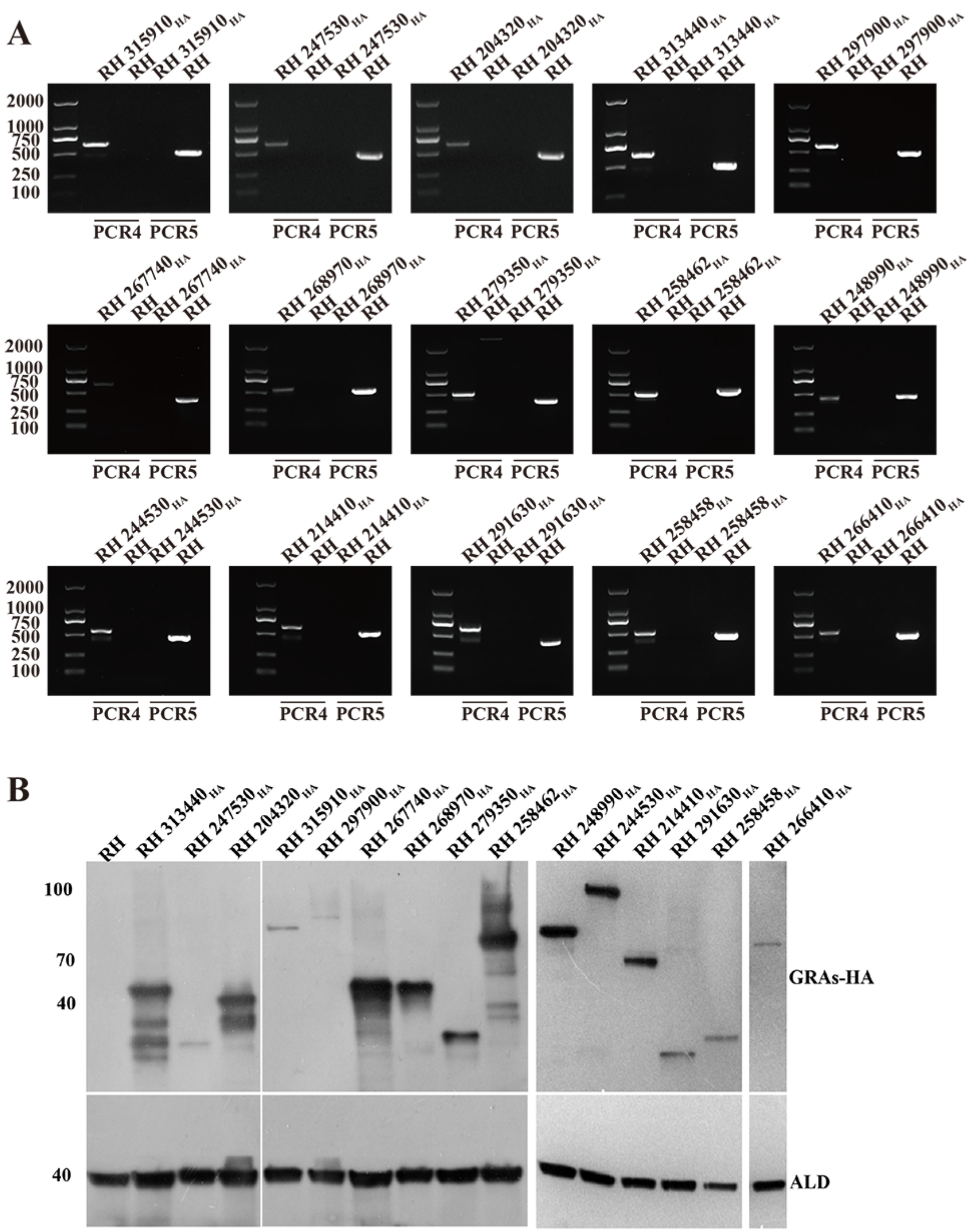

FIG S1 Construction of 15 *T. gondii* RH::*GRAs-HA* strains. **A** Identification of 15 RH::*GRAs-HA* strains by diagnostic PCRs. PCR4 was designed to detect the insert of six hemagglutinin (6×HA). PCR5 was designed to detect the successful replacement of C-terminal *gra* genes by 6×HA fragment. **B** Western blotting of 15 GRAs in RH strains, with aldolase (ALD) antibody as a loading control.

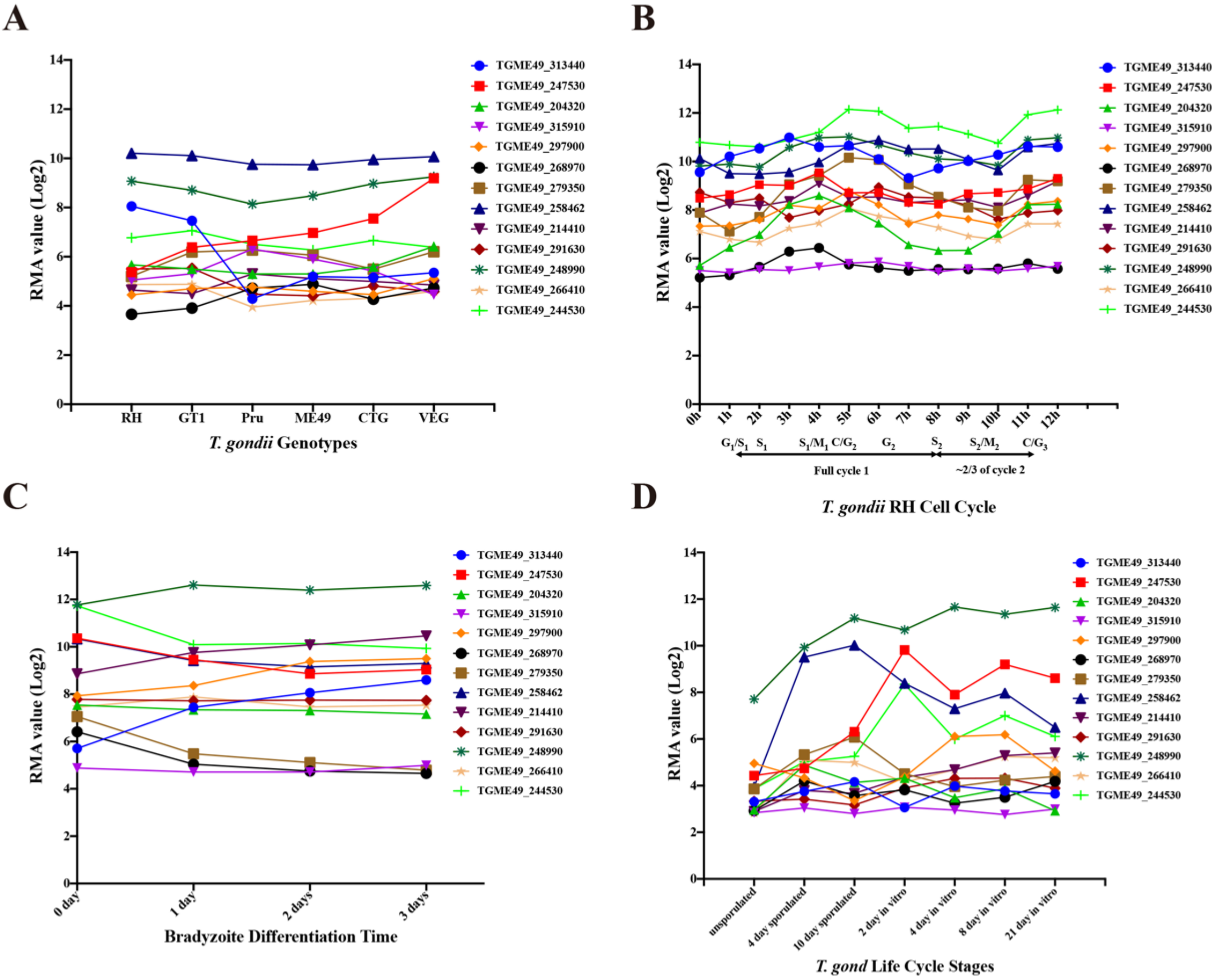

FIG S2 Expression profiles of *Toxoplasma gondii* GRAs. Transcriptional profiles of *gra* genes in strains of different genotypes (**A**) and different cell cycle phases of the RH strain (**B**), during bradyzoite differentiation (**C**) and in different life-cycle stages (**D**). The data was obtained from TOXODB and visualized as graphs with GraphPad Prism version 9.0.

Table S1 Primers used in the construction of RHΔ*gra*s.

Primer	Sequence (5′-3′)	Use
SgRNA-TGME49_313440-KO	GAGACGGTGGCCTCTCGCAG	SgRNA of the CRISPR plasmid for deleting *TGME49_313440*
SgRNA-TGME49_313440-KO-Fw	GAGACGGTGGCCTCTCGCAGGTTTTAGAGCTAGAAATAGC	Construct the CRISPR plasmid for deleting *TGME49_313440*
SgRNA-Rv	AACTTGACATCCCCATTTAC	Construct the CRISPR plasmid for deleting gra*s*
U5-TGME49_313440-Fw	GGTTTTCCCAGTCACGACGTTGAACACAGAAGCGATGAAC	Amplify the 5' homologous arms of *TGME49_313440* to construct the pUPRT-DHFR-D plasmid
U5-TGME49_313440-Rv	GGATTTACAGCCTGGCGAAGCTTGAGTGACATACCAGCGAAT	Amplify the 5' homologous arms of *TGME49_313440* to construct the pUPRT-DHFR-D plasmid
U3-TGME49_313440-Fw	CTATGCACTTGCAGGATGAATTCCCTCGTGACTGAATCTGA	Amplify the 3' homologous arms of *TGME49_313440* to construct the pUPRT-DHFR-D plasmid
U3-TGME49_313440-Rv	GAGCGGATAACAATTTCACACCAACATCTTCTGAGCATAT	Amplify the 3' homologous arms of *TGME49_313440* to construct the pUPRT-DHFR-D plasmid
DHFR-Fw	AAGCTTCGCCAGGCTGTAAATCC	Amplify the DHFR fragment to construct the pUPRT-DHFR-D plasmid
DHFR-Rv	GAATTCATCCTGCAAGTGCATAG	Amplify the DHFR fragment to construct the pUPRT-DHFR-D plasmid
pUC19-Fw	TGTGAAATTGTTATCCGCTC	Amplify the pUC19 fragment to construct the pUPRT-DHFR-D plasmid
pUC19-Rv	AACGTCGTGACTGGGAAAACC	Amplify the pUC19 fragment to construct the pUPRT-DHFR-D plasmid
TGME49_313440-KZ-Fw	GAACACAGAAGCGATGAAC	Amplify the 5UTR-DHFR-3UTR fragment of *TGME49_313440*
TGME49_313440-KZ-Rv	CCAACATCTTCTGAGCATAT	Amplify the 5UTR-DHFR-3UTR fragment of *TGME49_313440*
PCR1-TGME49_313440-Fw	AAGTCTGGAATCGATCGAGG	Detect the insertion of 5' homologous fragment of *TGME49_313440* in PCR1
PCR1-DHFR-Rv	GCCAAAGTAGAAAGGAATTAGCAT	Detect the insertion of 5' homologous fragment of *TGME49_313440* in PCR1
PCR2-TGME49_313440-Fw	AGGTCGCCACAGAAGAAT	Detect the deletion of *TGME49_313440* in PCR2
PCR2-TGME49_313440-Rv	TGCTTGTCTCCTCTCCAG	Detect the deletion of *TGME49_313440* in PCR2
PCR3-DHFR-Fw	TGACGCAGATGTGCGTGTATCCAC	Detect the insertion of 3' homologous fragment of *TGME49_313440* in PCR3
PCR3-TGME49_313440-Rv	GTAAGGCATTTCATTATCATGGCT	Detect the insertion of 3' homologous fragment of *TGME49_313440* in PCR3
SgRNA-TGME49_247530-KO	GCTTCGGTGTCAAGAATCTG	SgRNA of the CRISPR plasmid for deleting *TGME49_247530*
SgRNA-TGME49_247530-KO-Fw	GCTTCGGTGTCAAGAATCTGGTTTTAGAGCTAGAAATAGC	Construct the CRISPR plasmid for deleting *TGME49_247530*
U5-TGME49_247530-Fw	GGTTTTCCCAGTCACGACGTTTCAAGACACGGCAAGAAT	Amplify the 5' homologous arms of *TGME49_247530* to construct the pUPRT-DHFR-D plasmid
U5-TGME49_247530-Rv	GGATTTACAGCCTGGCGAAGCTTAAGAATCTGTGGTGAAGGT	Amplify the 5' homologous arms of *TGME49_247530* to construct the pUPRT-DHFR-D plasmid

U3-TGME49_247530-Fw	CTATGCACTTGCAGGATGAATTCTCAGCGAACCAACACAAT	Amplify the 3' homologous arms of *TGME49_247530* to construct the pUPRT-DHFR-D plasmid
U3-TGME49_247530-Rv	GAGCGGATAACAATTTCACATCAACGGCACAGTAGATAC	Amplify the 3' homologous arms of *TGME49_247530* to construct the pUPRT-DHFR-D plasmid
TGME49_247530-KZ-Fw	TCAAGACACGGCAAGAAT	Amplify the 5UTR-DHFR-3UTR fragment of *TGME49_247530*
TGME49_247530-KZ-Rv	TCAACGGCACAGTAGATAC	Amplify the 5UTR-DHFR-3UTR fragment of *TGME49_247530*
PCR1-TGME49_247530-Fw	GCGAGGCGTTTTCTATTTAGAAT	Detect the insertion of 5' homologous fragment of *TGME49_247530* in PCR1
PCR2-TGME49_247530-Fw	AAGTATGCCGTTCTTGTAGT	Detect the deletion of *TGME49_247530* in PCR2
PCR2-TGME49_247530-Rv	TTGTGTTGGTTCGCTGATA	Detect the deletion of *TGME49_247530* in PCR2
PCR3-TGME49_247530-Rv	GCATGCTACTCAATTAGGTAAAGAG	Detect the insertion of 3' homologous fragment of *TGME49_247530* in PCR3
SgRNA-TGME49_204320-KO	GGCCAGCAATATGAGAGCGG	SgRNA of the CRISPR plasmid for deleting *TGME49_204320*
SgRNA-TGME49_204320-KO-Fw	GGCCAGCAATATGAGAGCGGGTTTTAGAGCTAGAAATAGC	Construct the CRISPR plasmid for deleting *TGME49_204320*
U5-TGME49_204320-Fw	GGTTTTCCCAGTCACGACGTTGTTCTTCGTCGCTCCAAT	Amplify the 5' homologous arms of *TGME49_204320* to construct the pUPRT-DHFR-D plasmid
U5-TGME49_204320-Rv	GGATTTACAGCCTGGCGAAGCTTCCAAGACTCTCAGCAGTG	Amplify the 5' homologous arms of *TGME49_204320* to construct the pUPRT-DHFR-D plasmid
U3-TGME49_204320-Fw	CTATGCACTTGCAGGATGAATTCGAAGTGGTCTGATGGCATA	Amplify the 3' homologous arms of *TGME49_204320* to construct the pUPRT-DHFR-D plasmid
U3-TGME49_204320-Rv	GAGCGGATAACAATTTCACAGTTGACACAGATAGCGTTAG	Amplify the 3' homologous arms of *TGME49_204320* to construct the pUPRT-DHFR-D plasmid
TGME49_204320-KZ-Fw	GTTCTTCGTCGCTCCAAT	Amplify the 5UTR-DHFR-3UTR fragment of *TGME49_204320*
TGME49_204320-KZ-Rv	GTTGACACAGATAGCGTTAG	Amplify the 5UTR-DHFR-3UTR fragment of *TGME49_204320*
PCR1-TGME49_204320-Fw	CGCATGAAAGACTGAAATTGTTG	Detect the insertion of 5' homologous fragment of *TGME49_204320* in PCR1
PCR2-TGME49_204320-Fw	CGAACAACTATCTCCGTACA	Detect the deletion of *TGME49_204320* in PCR2
PCR2-TGME49_204320-Rv	TTGCTTGATCCGTCTCATT	Detect the deletion of *TGME49_204320* in PCR2
PCR3-TGME49_204320-Rv	AGAAACTTCCCACAAACCTTTG	Detect the insertion of 3' homologous fragment of *TGME49_204320* in PCR3
SgRNA-TGME49_266410-KO	GCAGGCGTTATCTTCGGTGC	SgRNA of the CRISPR plasmid for deleting *TGME49_266410*
SgRNA-TGME49_266410-KO-Fw	GCAGGCGTTATCTTCGGTGCGTTTTAGAGCTAGAAATAGC	Construct the CRISPR plasmid for deleting *TGME49_266410*
U5-TGME49_266410-Fw	GGTTTTCCCAGTCACGACGTTGCCGACTGTGGTTGTTAT	Amplify the 5' homologous arms of *TGME49_266410* to construct the pUPRT-DHFR-D plasmid
U5-TGME49_266410-Rv	GGATTTACAGCCTGGCGAAGCTTACTGACTCTGCGTAGGTT	Amplify the 5' homologous arms of *TGME49_266410* to construct the pUPRT-DHFR-D plasmid
U3-TGME49_266410-Fw	CTATGCACTTGCAGGATGAATTCCGAACGCCAAGATTGAAG	Amplify the 3' homologous arms of *TGME49_266410* to construct the pUPRT-DHFR-D plasmid

U3-TGME49_266410-Rv	GAGCGGATAACAATTTCACATTACACCAAGAGGAAGTAGG	Amplify the 3' homologous arms of *TGME49_266410* to construct the pUPRT-DHFR-D plasmid
TGME49_266410-KZ-Fw	GCCGACTGTGGTTGTTAT	Amplify the 5UTR-DHFR-3UTR fragment of *TGME49_266410*
TGME49_266410-KZ-Rv	TTACACCAAGAGGAAGTAGG	Amplify the 5UTR-DHFR-3UTR fragment of *TGME49_266410*
PCR1-TGME49_266410-Fw	TTTCGATTCGTAGGTAACGGAG	Detect the insertion of 5' homologous fragment of *TGME49_266410* in PCR1
PCR2-TGME49_266410-Fw	CGTGGAAGCCAACAGAAT	Detect the deletion of *TGME49_266410* in PCR2
PCR2-TGME49_266410-Rv	CTCCGATTCGCTCTTCAG	Detect the deletion of *TGME49_266410* in PCR2
PCR3-TGME49_266410-Rv	TCCCGTTTCTGTAAAGTTGTTCCTT	Detect the insertion of 3' homologous fragment of *TGME49_266410* in PCR3
SgRNA-TGME49_315910-KO	GAGCAGGAACAGAAGAGTGC	SgRNA of the CRISPR plasmid for deleting *TGME49_315910*
SgRNA-TGME49_315910-KO-Fw	GAGCAGGAACAGAAGAGTGCGTTTTAGAGCTAGAAATAGC	Construct the CRISPR plasmid for deleting *TGME49_315910*
U5-TGME49_315910-Fw	GGTTTTCCCAGTCACGACGTTTTGCCTTAGCCAGTGACAACTC	Amplify the 5' homologous arms of *TGME49_315910* to construct the pUPRT-DHFR-D plasmid
U5-TGME49_315910-Rv	GGATTTACAGCCTGGCGAAGCTTATCGACGCGATTCTACCGA	Amplify the 5' homologous arms of *TGME49_315910* to construct the pUPRT-DHFR-D plasmid
U3-TGME49_315910-Fw	CTATGCACTTGCAGGATGAATTCAAATGAAGACTCTGGAGGTGGG	Amplify the 3' homologous arms of *TGME49_315910* to construct the pUPRT-DHFR-D plasmid
U3-TGME49_315910-Rv	GAGCGGATAACAATTTCACACACATTTCCGTCAGCAATAACA	Amplify the 3' homologous arms of *TGME49_315910* to construct the pUPRT-DHFR-D plasmid
TGME49_315910-KZ-Fw	TTGCCTTAGCCAGTGACAACTC	Amplify the 5UTR-DHFR-3UTR fragment of *TGME49_315910*
TGME49_315910-KZ-Rv	CACATTTCCGTCAGCAATAACA	Amplify the 5UTR-DHFR-3UTR fragment of *TGME49_315910*
PCR1-TGME49_315910-Fw	TGGATGGAAGCTTTTTTGATTTCT	Detect the insertion of 5' homologous fragment of *TGME49_315910* in PCR1
PCR2-TGME49_315910-Fw	CCGCTTGGGTATCACTGGC	Detect the deletion of *TGME49_315910* in PCR2
PCR2-TGME49_315910-Rv	CGTCTTCGGCATCATTCTCC	Detect the deletion of *TGME49_315910* in PCR2
PCR3-TGME49_315910-Rv	AGCGGGTGAGTTCTGGAAAAA	Detect the insertion of 3' homologous fragment of *TGME49_315910* in PCR3
SgRNA-TGME49_297900-KO	GACACCGGCGCGTCTTCGCC	SgRNA of the CRISPR plasmid for deleting *TGME49_297900*
SgRNA-TGME49_297900-KO-Fw	GACACCGGCGCGTCTTCGCCGTTTTAGAGCTAGAAATAGC	Construct the CRISPR plasmid for deleting *TGME49_297900*
U5-TGME49_297900-Fw	GGTTTTCCCAGTCACGACGTTTTCGCAGAAGGCGGTGAT	Amplify the 5' homologous arms of *TGME49_297900* to construct the pUPRT-DHFR-D plasmid
U5-TGME49_297900-Rv	GGATTTACAGCCTGGCGAAGCTTCACGGCAGGGAACGTAAAA	Amplify the 5' homologous arms of *TGME49_297900* to construct the pUPRT-DHFR-D plasmid
U3-TGME49_297900-Fw	CTATGCACTTGCAGGATGAATTCGTGCTGGCGACTGACGAAT	Amplify the 3' homologous arms of *TGME49_297900* to construct the pUPRT-DHFR-D plasmid
U3-TGME49_297900-Rv	GAGCGGATAACAATTTCACAACCAACCGACTGAACGAACTT	Amplify the 3' homologous arms of *TGME49_297900* to construct the pUPRT-DHFR-D plasmid

TGME49_297900-KZ-Fw	TTCGCAGAAGGCGGTGAT	Amplify the 5UTR-DHFR-3UTR fragment of *TGME49_297900*
TGME49_297900-KZ-Rv	ACCAACCGACTGAACGAACTT	Amplify the 5UTR-DHFR-3UTR fragment of *TGME49_297900*
PCR1-TGME49_297900-Fw	TAGCACATAACTGGAAGGAGTATG	Detect the insertion of 5' homologous fragment of *TGME49_297900* in PCR1
PCR2-TGME49_297900-Fw	TAGCAAACGGACCAGACAAAAT	Detect the deletion of *TGME49_297900* in PCR2
PCR2-TGME49_297900-Rv	TGAGTCCCACGGGCAGAA	Detect the deletion of *TGME49_297900* in PCR2
PCR3-TGME49_297900-Rv	TTTCCCGTCAAGCGTTTTTG	Detect the insertion of 3' homologous fragment of *TGME49_297900* in PCR3
SgRNA-TGME49_267740-KO	GCTCGCCTCGCAATAACTGC	SgRNA of the CRISPR plasmid for deleting *TGME49_267740*
SgRNA-TGME49_267740-KO-Fw	GCTCGCCTCGCAATAACTGCGTTTTAGAGCTAGAAATAGC	Construct the CRISPR plasmid for deleting *TGME49_267740*
U5-TGME49_267740-Fw	GGTTTTCCCAGTCACGACGTTCCGCCTCGCCTTTGCCAACC	Amplify the 5' homologous arms of *TGME49_267740* to construct the pUPRT-DHFR-D plasmid
U5-TGME49_267740-Rv	GGATTTACAGCCTGGCGAAGCTTACACCGCCCGCCACCCTATC	Amplify the 5' homologous arms of *TGME49_267740* to construct the pUPRT-DHFR-D plasmid
U3-TGME49_267740-Fw	CTATGCACTTGCAGGATGAATTCCGGAACAAAGAGCACCCAG	Amplify the 3' homologous arms of *TGME49_267740* to construct the pUPRT-DHFR-D plasmid
U3-TGME49_267740-Rv	GAGCGGATAACAATTTCACAGTTAGTCGCAGACACGAGAAGG	Amplify the 3' homologous arms of *TGME49_267740* to construct the pUPRT-DHFR-D plasmid
TGME49_267740-KZ-Fw	CCGCCTCGCCTTTGCCAACC	Amplify the 5UTR-DHFR-3UTR fragment of *TGME49_267740*
TGME49_267740-KZ-Rv	GTTAGTCGCAGACACGAGAAGG	Amplify the 5UTR-DHFR-3UTR fragment of *TGME49_267740*
PCR1-TGME49_267740-Fw	TTCAAAGCAAAAACCCAGTCTTG	Detect the insertion of 5' homologous fragment of *TGME49_267740* in PCR1
PCR2-TGME49_267740-Fw	TGGCACCTTTCATAAAATCCC	Detect the deletion of *TGME49_267740* in PCR2
PCR2-TGME49_267740-Rv	TGTTGTTCACGTCCTCCTTCC	Detect the deletion of *TGME49_267740* in PCR2
PCR3-TGME49_267740-Rv	TACCAGCCACCCCTATTATAGT	Detect the insertion of 3' homologous fragment of *TGME49_267740* in PCR3
SgRNA-TGME49_268970-KO	GAGAGAAGTCTCAGTGCCGT	SgRNA of the CRISPR plasmid for deleting *TGME49_268970*
SgRNA-TGME49_268970-KO-Fw	GAGAGAAGTCTCAGTGCCGTGTTTTAGAGCTAGAAATAGC	Construct the CRISPR plasmid for deleting *TGME49_268970*
U5-TGME49_268970-Fw	GGTTTTCCCAGTCACGACGTTTTCTGTCTGGTCGCTCCCC	Amplify the 5' homologous arms of *TGME49_268970* to construct the pUPRT-DHFR-D plasmid
U5-TGME49_268970-Rv	GGATTTACAGCCTGGCGAAGCTTCAACGCTGCCCTCCCTTC	Amplify the 5' homologous arms of *TGME49_268970* to construct the pUPRT-DHFR-D plasmid
U3-TGME49_268970-Fw	CTATGCACTTGCAGGATGAATTCTCGCTAAGAACCCGAAGGTAG	Amplify the 3' homologous arms of *TGME49_268970* to construct the pUPRT-DHFR-D plasmid
U3-TGME49_268970-Rv	GAGCGGATAACAATTTCACATCAGACGGCGTGGACAATT	Amplify the 3' homologous arms of *TGME49_268970* to construct the pUPRT-DHFR-D plasmid
TGME49_268970-KZ-Fw	TTCTGTCTGGTCGCTCCCC	Amplify the 5UTR-DHFR-3UTR fragment of *TGME49_268970*

TGME49_268970-KZ-Rv	TCAGACGGCGTGGACAATT	Amplify the 5UTR-DHFR-3UTR fragment of *TGME49_268970*
PCR1-TGME49_268970-Fw	TTTCTCCCGTTGCATTTAGCAA	Detect the insertion of 5' homologous fragment of *TGME49_268970* in PCR1
PCR2-TGME49_268970-Fw	CGATGAACTTGCGAGGACG	Detect the deletion of *TGME49_268970* in PCR2
PCR2-TGME49_268970-Rv	TGCCAGTGCGGTAGATTTGTA	Detect the deletion of *TGME49_268970* in PCR2
PCR3-TGME49_268970-Rv	TGTTCACCCGTTCTTGGGAA	Detect the insertion of 3' homologous fragment of *TGME49_268970* in PCR3
SgRNA-TGME49_279350-KO	GCTCTTCAGGTGGCCAAGCC	SgRNA of the CRISPR plasmid for deleting *TGME49_279350*
SgRNA-TGME49_279350-KO-Fw	GCTCTTCAGGTGGCCAAGCCGTTTTAGAGCTAGAAATAGC	Construct the CRISPR plasmid for deleting *TGME49_279350*
U5-TGME49_279350-Fw	GGTTTTCCCAGTCACGACGTTGTGCTATGGTGCGGTGAGC	Amplify the 5' homologous arms of *TGME49_279350* to construct the pUPRT-DHFR-D plasmid
U5-TGME49_279350-Rv	GGATTTACAGCCTGGCGAAGCTTAAGAGGTACAGGTTCCGAGGG	Amplify the 5' homologous arms of *TGME49_279350* to construct the pUPRT-DHFR-D plasmid
U3-TGME49_279350-Fw	CTATGCACTTGCAGGATGAATTCGTTCAGTGTTGCTCCTTCTTGC	Amplify the 3' homologous arms of *TGME49_279350* to construct the pUPRT-DHFR-D plasmid
U3-TGME49_279350-Rv	GAGCGGATAACAATTTCACATGGAATCACCATACTCCCCTTG	Amplify the 3' homologous arms of *TGME49_279350* to construct the pUPRT-DHFR-D plasmid
TGME49_279350-KZ-Fw	GTGCTATGGTGCGGTGAGC	Amplify the 5UTR-DHFR-3UTR fragment of *TGME49_279350*
TGME49_279350-KZ-Rv	TGGAATCACCATACTCCCCTTG	Amplify the 5UTR-DHFR-3UTR fragment of *TGME49_279350*
PCR1-TGME49_279350-Fw	ATATTCTACGTTGTCTGTGAGGCTA	Detect the insertion of 5' homologous fragment of *TGME49_279350* in PCR1
PCR2-TGME49_279350-Fw	CTATTGCTTGCGGCTGTATTCT	Detect the deletion of *TGME49_279350* in PCR2
PCR2-TGME49_279350-Rv	CTCCCTCGGGTTCTGTTGG	Detect the deletion of *TGME49_279350* in PCR2
PCR3-TGME49_279350-Rv	CAACACTAGCCCTGTATTTTGTT	Detect the insertion of 3' homologous fragment of *TGME49_279350* in PCR3
SgRNA-TGME49_258462-KO	GGCAGGTCTGCTTCGTGGAG	SgRNA of the CRISPR plasmid for deleting *TGME49_258462*
SgRNA-TGME49_258462-KO-Fw	GGCAGGTCTGCTTCGTGGAGGTTTTAGAGCTAGAAATAGC	Construct the CRISPR plasmid for deleting *TGME49_258462*
U5-TGME49_258462-Fw	GGTTTTCCCAGTCACGACGTTCGGACGCAACTGATGACCT	Amplify the 5' homologous arms of *TGME49_258462* to construct the pUPRT-DHFR-D plasmid
U5-TGME49_258462-Rv	GGATTTACAGCCTGGCGAAGCTTCAGGCGTGGAAAGAGGGA	Amplify the 5' homologous arms of *TGME49_258462* to construct the pUPRT-DHFR-D plasmid
U3-TGME49_258462-Fw	CTATGCACTTGCAGGATGAATTCTTTGGAAGGCAAGGTCGC	Amplify the 3' homologous arms of *TGME49_258462* to construct the pUPRT-DHFR-D plasmid
U3-TGME49_258462-Rv	GAGCGGATAACAATTTCACAGTCGGCATTTGTGGGGTAA	Amplify the 3' homologous arms of *TGME49_258462* to construct the pUPRT-DHFR-D plasmid
TGME49_258462-KZ-Fw	CGGACGCAACTGATGACCT	Amplify the 5UTR-DHFR-3UTR fragment of *TGME49_258462*
TGME49_258462-KZ-Rv	GTCGGCATTTGTGGGGTAA	Amplify the 5UTR-DHFR-3UTR fragment of *TGME49_258462*

PCR1-TGME49_258462-Fw	TTTGCCTCAGAGGTAAGTGAC	Detect the insertion of 5' homologous fragment of *TGME49_258462* in PCR1
PCR2-TGME49_258462-Fw	GCCGAAAGGCGGTCTGTG	Detect the deletion of *TGME49_258462* in PCR2
PCR2-TGME49_258462-Rv	GCAGTCATACGCTCAAGAAACG	Detect the deletion of *TGME49_258462* in PCR2
PCR3-TGME49_258462-Rv	TTCATCCGAAAGGTATCAACTTTTT	Detect the insertion of 3' homologous fragment of *TGME49_258462* in PCR3
SgRNA-TGME49_248990-KO	GCGCTACGAGGCAACGGCCA	SgRNA of the CRISPR plasmid for deleting *TGME49_248990*
SgRNA-TGME49_248990-KO-Fw	GCGCTACGAGGCAACGGCCAGTTTTAGAGCTAGAAATAGC	Construct the CRISPR plasmid for deleting *TGME49_248990*
U5-TGME49_248990-Fw	GGTTTTCCCAGTCACGACGTTCCGCTCAAGGAGAAACCG	Amplify the 5' homologous arms of *TGME49_248990* to construct the pUPRT-DHFR-D plasmid
U5-TGME49_248990-Rv	GGATTTACAGCCTGGCGAAGCTTACAAGGAAACCCGTGACCAT	Amplify the 5' homologous arms of *TGME49_248990* to construct the pUPRT-DHFR-D plasmid
U3-TGME49_248990-Fw	CTATGCACTTGCAGGATGAATTCTGCTCAGGCGTTCAATCTCA	Amplify the 3' homologous arms of *TGME49_248990* to construct the pUPRT-DHFR-D plasmid
U3-TGME49_248990-Rv	GAGCGGATAACAATTTCACACAACTCGACTGCCGTGTCTTAC	Amplify the 3' homologous arms of *TGME49_248990* to construct the pUPRT-DHFR-D plasmid
TGME49_248990-KZ-Fw	CCGCTCAAGGAGAAACCG	Amplify the 5UTR-DHFR-3UTR fragment of *TGME49_248990*
TGME49_248990-KZ-Rv	CAACTCGACTGCCGTGTCTTAC	Amplify the 5UTR-DHFR-3UTR fragment of *TGME49_248990*
PCR1-TGME49_248990-Fw	CTCTCTATTCTCTCGCGTAGCC	Detect the insertion of 5' homologous fragment of *TGME49_248990* in PCR1
PCR2-TGME49_248990-Fw	AACAAGGCGGCTGCTGAT	Detect the deletion of *TGME49_248990* in PCR2
PCR2-TGME49_248990-Rv	GTGACCCCACTCATAAAATCCA	Detect the deletion of *TGME49_248990* in PCR2
PCR3-TGME49_248990-Rv	GACCCAACGAAGAAAAGTCTGG	Detect the insertion of 3' homologous fragment of *TGME49_248990* in PCR3
SgRNA-TGME49_244530-KO	GCTGAAGCTAACTCGGACGC	SgRNA of the CRISPR plasmid for deleting *TGME49_244530*
SgRNA-TGME49_244530-KO-Fw	GCTGAAGCTAACTCGGACGCGTTTTAGAGCTAGAAATAGC	Construct the CRISPR plasmid for deleting *TGME49_244530*
U5-TGME49_244530-Fw	GGTTTTCCCAGTCACGACGTTCACCGCCAATGAACAAAAGC	Amplify the 5' homologous arms of *TGME49_244530* to construct the pUPRT-DHFR-D plasmid
U5-TGME49_244530-Rv	GGATTTACAGCCTGGCGAAGCTTGCAGGAAACCGTTGAGGGA	Amplify the 5' homologous arms of *TGME49_244530* to construct the pUPRT-DHFR-D plasmid
U3-TGME49_244530-Fw	CTATGCACTTGCAGGATGAATTCCGGCGTACCGTCACTTCAA	Amplify the 3' homologous arms of *TGME49_244530* to construct the pUPRT-DHFR-D plasmid
U3-TGME49_244530-Rv	GAGCGGATAACAATTTCACATTCGCATAGTCTGTTTACACTAGCA	Amplify the 3' homologous arms of *TGME49_244530* to construct the pUPRT-DHFR-D plasmid
TGME49_244530-KZ-Fw	CACCGCCAATGAACAAAAGC	Amplify the 5UTR-DHFR-3UTR fragment of *TGME49_244530*
TGME49_244530-KZ-Rv	TTCGCATAGTCTGTTTACACTAGCA	Amplify the 5UTR-DHFR-3UTR fragment of *TGME49_244530*
PCR1-TGME49_244530-Fw	GGCACAAAAACAAAAAGACGGGA	Detect the insertion of 5' homologous fragment of *TGME49_244530* in PCR1

PCR2-TGME49_244530-Fw	CCCCTGTCTGTGCATGGTTC	Detect the deletion of *TGME49_244530* in PCR2
PCR2-TGME49_244530-Rv	GTCTTCCCCGTCCGTGTTC	Detect the deletion of *TGME49_244530* in PCR2
PCR3-TGME49_244530-Rv	GGCAAATGCGTCATACCCAAC	Detect the insertion of 3' homologous fragment of *TGME49_244530* in PCR3
SgRNA-TGME49_214410-KO	GCGACCGCACAATGGTGAAC	SgRNA of the CRISPR plasmid for deleting *TGME49_214410*
SgRNA-TGME49_214410-KO-Fw	GCGACCGCACAATGGTGAACGTTTTAGAGCTAGAAATAGC	Construct the CRISPR plasmid for deleting *TGME49_214410*
U5-TGME49_214410-Fw	GGTTTTCCCAGTCACGACGTTTCGTTGCTGTTCGTTGACTTCG	Amplify the 5' homologous arms of *TGME49_214410* to construct the pUPRT-DHFR-D plasmid
U5-TGME49_214410-Rv	GGATTTACAGCCTGGCGAAGCTTGCGCCATCACGGCACTCT	Amplify the 5' homologous arms of *TGME49_214410* to construct the pUPRT-DHFR-D plasmid
U3-TGME49_214410-Fw	CTATGCACTTGCAGGATGAATTCAGCATTCCCGACGAGCCT	Amplify the 3' homologous arms of *TGME49_214410* to construct the pUPRT-DHFR-D plasmid
U3-TGME49_214410-Rv	GAGCGGATAACAATTTCACAGAAATCCGTTTTCCCTGTTTCACAA	Amplify the 3' homologous arms of *TGME49_214410* to construct the pUPRT-DHFR-D plasmid
TGME49_214410-KZ-Fw	TCGTTGCTGTTCGTTGACTTCG	Amplify the 5UTR-DHFR-3UTR fragment of *TGME49_214410*
TGME49_214410-KZ-Rv	GAAATCCGTTTTCCCTGTTTCACAA	Amplify the 5UTR-DHFR-3UTR fragment of *TGME49_214410*
PCR1-TGME49_214410-Fw	ATAACCAAGGAAGAGGGGGTTCT	Detect the insertion of 5' homologous fragment of *TGME49_214410* in PCR1
PCR2-TGME49_214410-Fw	ACCACATCGTGGTGGACAGG	Detect the deletion of *TGME49_214410* in PCR2
PCR2-TGME49_214410-Rv	ATGAATCGTTAGCAGGGAGCGGCAT	Detect the deletion of *TGME49_214410* in PCR2
PCR3-TGME49_214410-Rv	AAGCTCCAGACCCTAATCTGATAC	Detect the insertion of 3' homologous fragment of *TGME49_214410* in PCR3
SgRNA-TGME49_291630-KO	GGAGGATGCCGTGTCGAGTC	SgRNA of the CRISPR plasmid for deleting *TGME49_291630*
SgRNA-TGME49_291630-KO-Fw	GGAGGATGCCGTGTCGAGTCGTTTTAGAGCTAGAAATAGC	Construct the CRISPR plasmid for deleting *TGME49_291630*
U5-TGME49_291630-Fw	GGTTTTCCCAGTCACGACGTTGCAAGAAATGACGCGGCTAT	Amplify the 5' homologous arms of *TGME49_291630* to construct the pUPRT-DHFR-D plasmid
U5-TGME49_291630-Rv	GGATTTACAGCCTGGCGAAGCTTCGACACGGCATCCTCCAA	Amplify the 5' homologous arms of *TGME49_291630* to construct the pUPRT-DHFR-D plasmid
U3-TGME49_291630-Fw	CTATGCACTTGCAGGATGAATTCGAAGTGGGAACGACGCTGAA	Amplify the 3' homologous arms of *TGME49_291630* to construct the pUPRT-DHFR-D plasmid
U3-TGME49_291630-Rv	GAGCGGATAACAATTTCACAAGTGCTGGGCGGATGTCA	Amplify the 3' homologous arms of *TGME49_291630* to construct the pUPRT-DHFR-D plasmid
TGME49_291630-KZ-Fw	GCAAGAAATGACGCGGCTAT	Amplify the 5UTR-DHFR-3UTR fragment of *TGME49_291630*
TGME49_291630-KZ-Rv	AGTGCTGGGCGGATGTCA	Amplify the 5UTR-DHFR-3UTR fragment of *TGME49_291630*
PCR1-TGME49_291630-Fw	GTAATCACCACGGCACTAGGAG	Detect the insertion of 5' homologous fragment of *TGME49_291630* in PCR1
PCR2-TGME49_291630-Fw	GGACCCTCTTCGTTTTGTGC	Detect the deletion of *TGME49_291630* in PCR2

PCR2-TGME49_291630-Rv	TCAGCGTCGTTCCCACTTC	Detect the deletion of *TGME49_291630* in PCR2
PCR3-TGME49_291630-Rv	GCATGTCTCCCGTTTGTCTTC	Detect the insertion of 3' homologous fragment of *TGME49_291630* in PCR3
SgRNA-TGME49_258458-KO	GGTGATACTCCAGAGTAAGG	SgRNA of the CRISPR plasmid for deleting TGME49_258458
SgRNA-TGME49_258458-KO-Fw	GGTGATACTCCAGAGTAAGGGTTTTAGAGCTAGAAATAGC	Construct the CRISPR plasmid for deleting TGME49_258458
U5-TGME49_258458-Fw	GGTTTTCCCAGTCACGACGTTACAGCTACACCTACAAGCCACG	Amplify the 5' homologous arms of *TGME49_258458* to construct the pUPRT-DHFR-D plasmid
U5-TGME49_258458-Rv	GGATTTACAGCCTGGCGAAGCTTAAAAGCCAGTGAAAATGGAACA	Amplify the 5' homologous arms of *TGME49_258458* to construct the pUPRT-DHFR-D plasmid
U3-TGME49_258458-Fw	CTATGCACTTGCAGGATGAATTCGACTGTCTTGGTGCCTCGGTA	Amplify the 3' homologous arms of *TGME49_258458* to construct the pUPRT-DHFR-D plasmid
U3-TGME49_258458-Rv	GAGCGGATAACAATTTCACAGCTGGCTGAGCCTAAACTGG	Amplify the 3' homologous arms of *TGME49_258458* to construct the pUPRT-DHFR-D plasmid
TGME49_258458-KZ-Fw	ACAGCTACACCTACAAGCCACG	Amplify the 5UTR-DHFR-3UTR fragment of *TGME49_258458*
TGME49_258458-KZ-Rv	GCTGGCTGAGCCTAAACTGG	Amplify the 5UTR-DHFR-3UTR fragment of *TGME49_258458*
PCR1-TGME49_258458-Fw	CTCGGCAGAAAGAATGCTGAAG	Detect the insertion of 5' homologous fragment of *TGME49_258458* in PCR1
PCR2-TGME49_258458-Fw	GGGTTCGGGTCCTCTTTGA	Detect the deletion of *TGME49_258458* in PCR2
PCR2-TGME49_258458-Rv	TGGGAATTGGTGTTCCTTTATG	Detect the deletion of *TGME49_258458* in PCR2
PCR3-TGME49_258458-Rv	CAAGACTTGCCCTGCTTTTCT	Detect the insertion of 3' homologous fragment of *TGME49_258458* in PCR3

Table S2 Primers used in the construction of epitope-tagging strains.

Primer	Sequence (5′-3′)	Use
SgRNA-TGME49_313440-Tag	TTCCCTAATCGTCTAGCGCG	SgRNA of the CRISPR plasmid for tagging TGME49_313440 with 6×HA
SgRNA-TGME49_313440-Tag-Fw	TTCCCTAATCGTCTAGCGCGGTTTTAGAGCTAGAAATAGC	Construct the CRISPR plasmid for tagging TGME49_313440 with 6×HA
SgRNA-Rv	AACTTGACATCCCCATTTAC	Construct the CRISPR plasmid for tagging GRAs
TGME49_313440-HRF	TCTTCAGCCCCGACCGCGCAACCAACGGGTACCCCTCGTGACGCTAGCAAGGGCTCGGG	Amplify 6HA-DHFR fragment with the homologous arms of *TGME49_313440*
TGME49_313440-HRR	GTATCTCCAGCCACTTCGAGGAAATATTGACTGACACCGCGCATACGACTCACTATAGG	Amplify 6HA-DHFR fragment with the homologous arms of *TGME49_313440*
PCR4-Flag-Rv	ATTATACCCGTGTGTTACG	Detect the insert 6×HA fragment in PCR4
PCR4/PCR5-TGME49_313440-Fw	AGCCTTTTGGAGCGACTTCCT	Detect the insert 6×HA fragment in PCR4<br>Detect the replacement of C-terminal *TGME49_313440* by 6×HA fragment in PCR5
PCR5-TGME49_313440-Rv	TGAGTTTCGGTTCCTGGTTGG	Detect the replacement of C-terminal *TGME49_313440* by 6×HA fragment in PCR5
SgRNA-TGME49_247530-Tag	GAAAGCCTTCGAGGGTTATC	SgRNA of the CRISPR plasmid for tagging TGME49_247530 with 6×HA
SgRNA-TGME49_247530-Tag-Fw	GAAAGCCTTCGAGGGTTATCGTTTTAGAGCTAGAAATAGC	Construct the CRISPR plasmid for tagging TGME49_247530 with 6×HA
TGME49_247530-HRF	GCGCAGAGCACCTTGTCCACTTACCAGGCAAGGGATCACGACGCTAGCAAGGGCTCGGG	Amplify 6HA-DHFR fragment with the homologous arms of *TGME49_247530*
TGME49_247530-HRR	GCTTTGTACGTGCCCACGACGCGACACCGCTTCTCTCCTGATATACGACTCACTATAGG	Amplify 6HA-DHFR fragment with the homologous arms of *TGME49_247530*
PCR4/PCR5-TGME49_247530-Fw	CCGAAGCATTACAGGAAGCAA	Detect the insert 6×HA fragment in PCR4<br>Detect the replacement of C-terminal *TGME49_247530* by 6×HA fragment in PCR5
PCR5-TGME49_247530-Rv	TGTCACAAACGAATACAAGAGCC	Detect the replacement of C-terminal *TGME49_247530* by 6×HA fragment in PCR5
SgRNA-TGME49_204320-Tag	AAAGTAGTGTAGCGGTAGTG	SgRNA of the CRISPR plasmid for tagging TGME49_204320 with 6×HA
SgRNA-TGME49_204320-Tag-Fw	AAAGTAGTGTAGCGGTAGTGGTTTTAGAGCTAGAAATAGC	Construct the CRISPR plasmid for tagging TGME49_204320 with 6×HA
TGME49_204320-HRF	AAGAAGTGGTCTGATGGCATATTGTCAAACAGAAAACGAAAGGCTAGCAAGGGCTCGGG	Amplify 6HA-DHFR fragment with the homologous arms of *TGME49_204320*
TGME49_204320-HRR	AGACAAAATGTGGCTGCCGGCGAACAGGAAGCAGTGCCTCACATACGACTCACTATAGG	Amplify 6HA-DHFR fragment with the homologous arms of *TGME49_204320*
PCR4/PCR5-TGME49_204320-Fw	CAAGCAAACCGTTTACACCCC	Detect the insert 6×HA fragment in PCR4<br>Detect the replacement of C-terminal *TGME49_204320* by 6×HA fragment in PCR5
PCR5-TGME49_204320-Rv	CGTTCGTCAAGCCTTAGTTCTACAT	Detect the replacement of C-terminal *TGME49_204320* by 6×HA fragment in PCR5
SgRNA-TGME49_266410-Tag	GGGATGCAGATCGACTACGA	SgRNA of the CRISPR plasmid for tagging TGME49_266410 with 6×HA
SgRNA-TGME49_266410-Tag-Fw	GGGATGCAGATCGACTACGAGTTTTAGAGCTAGAAATAGC	Construct the CRISPR plasmid for tagging TGME49_266410 with 6×HA
TGME49_266410-HRF	GACCCGCAGCAGCTGCGGGCTGCGATCCAGGCGCAGGAAGCGGCTAGCAAGGGCTCGGG	Amplify 6HA-DHFR fragment with the homologous arms of *TGME49_266410*

TGME49_266410-HRR	TCCTGGTTTGAAGTGAACCTGGTCAGGAGTGTTCGACCTTCGATACGACTCACTATAGG	Amplify 6HA-DHFR fragment with the homologous arms of *TGME49_266410*
PCR4/PCR5-TGME49_266410-Fw	GCCGCAGAAGCAACCAAAC	Detect the insert 6×HA fragment in PCR4<br>Detect the replacement of C-terminal *TGME49_266410* by 6×HA fragment in PCR5
PCR5-TGME49_266410-Rv	GGTCCGTCTCAACCGCTTATCT	Detect the replacement of C-terminal *TGME49_266410* by 6×HA fragment in PCR5
SgRNA-TGME49_315910-Tag	CGTAAGAGCTTCTCCTTTCG	SgRNA of the CRISPR plasmid for tagging TGME49_315910 with 6×HA
SgRNA-TGME49_315910-Tag-Fw	CGTAAGAGCTTCTCCTTTCGGTTTTAGAGCTAGAAATAGC	Construct the CRISPR plasmid for tagging TGME49_315910 with 6×HA
TGME49_315910-HRF	GGCGACGAGGATGAAGAGGGAGCGCAGGAGGCGGAGTCCGCGGCTAGCAAGGGCTCGG<br>G	Amplify 6HA-DHFR fragment with the homologous arms of *TGME49_315910*
TGME49_315910-HRR	TGAAGAATCATTCGGAAGTGGATGCTGCCCAGTGCGCCTCGAATACGACTCACTATAGG	Amplify 6HA-DHFR fragment with the homologous arms of *TGME49_315910*
PCR4/PCR5-TGME49_315910-Fw	CTCTGGAGGTGGGAGACTTGG	Detect the insert 6×HA fragment in PCR4<br>Detect the replacement of C-terminal *TGME49_315910* by 6×HA fragment in PCR5
PCR5-TGME49_315910-Rv	CATCGGGCGGACGGTTTT	Detect the replacement of C-terminal *TGME49_315910* by 6×HA fragment in PCR5
SgRNA-TGME49_297900-Tag	GAAACAGCGGGGGACAATGC	SgRNA of the CRISPR plasmid for tagging TGME49_297900 with 6×HA
SgRNA-TGME49_297900-Tag-Fw	GAAACAGCGGGGGACAATGCGTTTTAGAGCTAGAAATAGC	Construct the CRISPR plasmid for tagging TGME49_297900 with 6×HA
TGME49_297900-HRF	ACGTTTTTTGCTGCTTTGGGATATTTGGTGAATTCTGCGGTAGCTAGCAAGGGCTCGGG	Amplify 6HA-DHFR fragment with the homologous arms of *TGME49_297900*
TGME49_297900-HRR	AGAGTTCAAAAGAATTTACCACGAAACGGGTCTTGTCCCGCAATACGACTCACTATAGG	Amplify 6HA-DHFR fragment with the homologous arms of *TGME49_297900*
PCR4/PCR5-TGME49_297900-Fw	CCCCGTATCTCGTCCACATC	Detect the insert 6×HA fragment in PCR4<br>Detect the replacement of C-terminal *TGME49_297900* by 6×HA fragment in PCR5
PCR5-TGME49_297900-Rv	AACTCGGTTGACAAACACTTCG	Detect the replacement of C-terminal *TGME49_297900* by 6×HA fragment in PCR5
SgRNA-TGME49_267740-Tag	GATTGATCGAGATACAACTC	SgRNA of the CRISPR plasmid for tagging TGME49_267740 with 6×HA
SgRNA-TGME49_267740-Tag-Fw	GATTGATCGAGATACAACTCGTTTTAGAGCTAGAAATAGC	Construct the CRISPR plasmid for tagging TGME49_267740 with 6×HA
TGME49_267740-HRF	GAACTGGATAAGCAGACTGCCTTCGGCACCTCACTTGATGACGCTAGCAAGGGCTCGGG	Amplify 6HA-DHFR fragment with the homologous arms of *TGME49_267740*
TGME49_267740-HRR	CACGATCCAGCTCGACTCTCAGGAACGGAATGGCCTCCCGAGATACGACTCACTATAGG	Amplify 6HA-DHFR fragment with the homologous arms of *TGME49_267740*
PCR4/PCR5-TGME49_267740-Fw	GGAGTGGGATCAACTATGTGGG	Detect the insert 6×HA fragment in PCR4<br>Detect the replacement of C-terminal *TGME49_267740* by 6×HA fragment in PCR5
PCR5-TGME49_267740-Rv	TGACGGGGAACGCTACGA	Detect the replacement of C-terminal *TGME49_267740* by 6×HA fragment in PCR5
SgRNA-TGME49_268970-Tag	AAGCAGTAGCTGAATATCTA	SgRNA of the CRISPR plasmid for tagging TGME49_268970 with 6×HA
SgRNA-TGME49_268970-Tag-Fw	AAGCAGTAGCTGAATATCTAGTTTTAGAGCTAGAAATAGC	Construct the CRISPR plasmid for tagging TGME49_268970 with 6×HA
TGME49_268970-HRF	GGCGAGTATAAATCCTCTGCATCAGATGATGACACTAAGCAGGCTAGCAAGGGCTCGGG	Amplify 6HA-DHFR fragment with the homologous arms of *TGME49_268970*
TGME49_268970-HRR	CCGCAGCGTGAACTCGAGTCCCTTCTGAGTAAATCGCCGTAGATACGACTCACTATAGG	Amplify 6HA-DHFR fragment with the homologous arms of *TGME49_268970*

PCR4/PCR5-TGME49_268970-Fw	GCACTGGCAAAAGCAGGAG	Detect the insert 6×HA fragment in PCR4<br>Detect the replacement of C-terminal *TGME49_268970* by 6×HA fragment in PCR5
PCR5-TGME49_268970-Rv	CTCTTTACAAAGCATTTCGGGCATA	Detect the replacement of C-terminal *TGME49_268970* by 6×HA fragment in PCR5
SgRNA-TGME49_279350-Tag	TCTTGTGGAACTGTGTGAAA	SgRNA of the CRISPR plasmid for tagging TGME49_279350 with 6×HA
SgRNA-TGME49_279350-Tag-Fw	TCTTGTGGAACTGTGTGAAAGTTTTAGAGCTAGAAATAGC	Construct the CRISPR plasmid for tagging TGME49_279350 with 6×HA
TGME49_279350-HRF	GTTGCTCCTTCTTGCGATTGCTCGGTATCTTGTGGAACTGTGGCTAGCAAGGGCTCGGG	Amplify 6HA-DHFR fragment with the homologous arms of *TGME49_279350*
TGME49_279350-HRR	AATGAAGAACCATGAGGCTGAAATGAAAGAACGTTCCCCTTTATACGACTCACTATAGG	Amplify 6HA-DHFR fragment with the homologous arms of *TGME49_279350*
PCR4/PCR5-TGME49_279350-Fw	CCGCAGGGATGGAAAAGC	Detect the insert 6×HA fragment in PCR4<br>Detect the replacement of C-terminal *TGME49_279350* by 6×HA fragment in PCR5
PCR5-TGME49_279350-Rv	TGCTCGGCTGCCAAACAC	Detect the replacement of C-terminal *TGME49_279350* by 6×HA fragment in PCR5
SgRNA-TGME49_258462-Tag	GATGATACCTTATGAATGAA	SgRNA of the CRISPR plasmid for tagging TGME49_258462 with 6×HA
SgRNA-TGME49_258462-Tag-Fw	GATGATACCTTATGAATGAAGTTTTAGAGCTAGAAATAGC	Construct the CRISPR plasmid for tagging TGME49_258462 with 6×HA
TGME49_258462-HRF	GCGTCCAAGTTACGAGTTGTTTTTATTGTTGATGATACCTTAGCTAGCAAGGGCTCGGG	Amplify 6HA-DHFR fragment with the homologous arms of *TGME49_258462*
TGME49_258462-HRR	CACAGAGGCTTGGAAATTACCTGACCACACGAAAGCCCCTTCATACGACTCACTATAGG	Amplify 6HA-DHFR fragment with the homologous arms of *TGME49_258462*
PCR4/PCR5-TGME49_258462-Fw	GGGTTCTGGGCGTTTCTGT	Detect the insert 6×HA fragment in PCR4<br>Detect the replacement of C-terminal *TGME49_258462* by 6×HA fragment in PCR5
PCR5-TGME49_258462-Rv	GCTGTATTTTGCGAGCACTCTT	Detect the replacement of C-terminal *TGME49_258462* by 6×HA fragment in PCR5
SgRNA-TGME49_248990-Tag	GAAGTGTAAACGGCGTGGAG	SgRNA of the CRISPR plasmid for tagging TGME49_248990 with 6×HA
SgRNA-TGME49_248990-Tag-Fw	GAAGTGTAAACGGCGTGGAGGTTTTAGAGCTAGAAATAGC	Construct the CRISPR plasmid for tagging TGME49_248990 with 6×HA
TGME49_248990-HRF	AACCTGATCAGTAACAGTCTCGACATATACTCAGGTGAAGTGGCTAGCAAGGGCTCGGG	Amplify 6HA-DHFR fragment with the homologous arms of *TGME49_248990*
TGME49_248990-HRR	GGATCGAGCGCGCACACAGAGTAGTAAACGTCGGCACCTCTCATACGACTCACTATAGG	Amplify 6HA-DHFR fragment with the homologous arms of *TGME49_248990*
PCR4/PCR5-TGME49_248990-Fw	TTCTACCCAAGTGCATTTGAATC	Detect the insert 6×HA fragment in PCR4<br>Detect the replacement of C-terminal *TGME49_248990* by 6×HA fragment in PCR5
PCR5-TGME49_248990-Rv	GACCGACTACCTGCGTGTTTT	Detect the replacement of C-terminal *TGME49_248990* by 6×HA fragment in PCR5
SgRNA-TGME49_244530-Tag	GAGAAGATTGTGTGCCAAAC	SgRNA of the CRISPR plasmid for tagging TGME49_244530 with 6×HA
SgRNA-TGME49_244530-Tag-Fw	GAGAAGATTGTGTGCCAAACGTTTTAGAGCTAGAAATAGC	Construct the CRISPR plasmid for tagging TGME49_244530 with 6×HA
TGME49_244530-HRF	CAAGTGGAGGTTCCTACTGAAACTGGCGTCTCCCACGGTGAGGCTAGCAAGGGCTCGGG	Amplify 6HA-DHFR fragment with the homologous arms of *TGME49_244530*
TGME49_244530-HRR	CGTGAACCAAGGAGCTGGCTATGCCCGAGACACCGGCCCGTTATACGACTCACTATAGG	Amplify 6HA-DHFR fragment with the homologous arms of *TGME49_244530*
PCR4/PCR5-TGME49_244530-Fw	GAATGCCGTGGAAGAACCTG	Detect the insert 6×HA fragment in PCR4<br>Detect the replacement of C-terminal *TGME49_244530* by 6×HA fragment in PCR5

PCR5-TGME49_244530-Rv	CTACGCTTTTAGCAGTCGAGGA	Detect the replacement of C-terminal *TGME49_244530* by 6×HA fragment in PCR5
SgRNA-TGME49_214410-Tag	CCTTGAATTATTTTCTACAT	SgRNA of the CRISPR plasmid for tagging TGME49_214410 with 6×HA
SgRNA-TGME49_214410-Tag-Fw	CCTTGAATTATTTTCTACATGTTTTAGAGCTAGAAATAGC	Construct the CRISPR plasmid for tagging TGME49_214410 with 6×HA
TGME49_214410-HRF	TTGCAGAGCATTCCCGACGAGCCTGGTGACGGAGAAATTCCTGCTAGCAAGGGCTCGGG	Amplify 6HA-DHFR fragment with the homologous arms of *TGME49_214410*
TGME49_214410-HRR	TGTCGCCGCACTGTGGCTCAAGGAAGACCTGCGAACCCGATGATACGACTCACTATAGG	Amplify 6HA-DHFR fragment with the homologous arms of *TGME49_214410*
PCR4/PCR5-TGME49_214410-Fw	TCGACCGTTGGTAGCATAGC	Detect the insert 6×HA fragment in PCR4<br>Detect the replacement of C-terminal *TGME49_214410* by 6×HA fragment in PCR5
PCR5-TGME49_214410-Rv	CGTTGACAAAATACTCGCCTCT	Detect the replacement of C-terminal *TGME49_214410* by 6×HA fragment in PCR5
SgRNA-TGME49_291630-Tag	GCAGCAATCGCCTTGTTATA	SgRNA of the CRISPR plasmid for tagging TGME49_291630 with 6×HA
SgRNA-TGME49_291630-Tag-Fw	GCAGCAATCGCCTTGTTATAGTTTTAGAGCTAGAAATAGC	Construct the CRISPR plasmid for tagging TGME49_291630 with 6×HA
TGME49_291630-HRF	AAGGACCAGGGCATCCAGATTGAGCTGAGAAAAGGCTCGATTGCTAGCAAGGGCTCGGG	Amplify 6HA-DHFR fragment with the homologous arms of *TGME49_291630*
TGME49_291630-HRR	TAGAGGCGCCTGCTCAATGCATGCGCGTCTTCAGGTCCTTATATACGACTCACTATAGG	Amplify 6HA-DHFR fragment with the homologous arms of *TGME49_291630*
PCR4/PCR5-TGME49_291630-Fw	GCTGGCGGTTGTTCTGGG	Detect the insert 6×HA fragment in PCR4<br>Detect the replacement of C-terminal *TGME49_291630* by 6×HA fragment in PCR5
PCR5-TGME49_291630-Rv	GCATCTGCCTTCGGCTGTC	Detect the replacement of C-terminal *TGME49_291630* by 6×HA fragment in PCR5
SgRNA-TGME49_258458-Tag	TAGTTGTGAATCTCCCGACG	SgRNA of the CRISPR plasmid for tagging TGME49_258458 with 6×HA
SgRNA-TGME49_258458-Tag-Fw	TAGTTGTGAATCTCCCGACGGTTTTAGAGCTAGAAATAGC	Construct the CRISPR plasmid for tagging TGME49_258458 with 6×HA
TGME49_258458-HRF	CAAGAATGGCATGACGACTCGACTTGCTGCCCCGTGAACAGAGCTAGCAAGGGCTCGGG	Amplify 6HA-DHFR fragment with the homologous arms of *TGME49_258458*
TGME49_258458-HRR	CATGAGGCCAGGTAGAGTCTCTCCCTGAATGGAAGACCACGTATACGACTCACTATAGG	Amplify 6HA-DHFR fragment with the homologous arms of *TGME49_258458*
PCR4/PCR5-TGME49_258458-Fw	GATCCGCTTCGTGTAGATTCATT	Detect the insert 6×HA fragment in PCR4<br>Detect the replacement of C-terminal *TGME49_258458* by 6×HA fragment in PCR5
PCR5-TGME49_258458-Rv	AACTGTCGCCGCCGTGT	Detect the replacement of C-terminal *TGME49_258458* by 6×HA fragment in PCR5
